# Effects of Primary Viruses (PCV2, PPV1, and PRRSV) Involved in Porcine Reproductive Failure as Mono- and Coinfections with Each Other and with Emerging Viruses (PCV3 and nPPVs)

**DOI:** 10.3390/v17081137

**Published:** 2025-08-19

**Authors:** Diana S. Vargas-Bermudez, Jose Dario Mogollon, Jairo Jaime

**Affiliations:** Universidad Nacional de Colombia, Sede Bogotá, Facultad de Medicina Veterinaria y de Zootecnia, Departamento de Salud Animal, Centro de Investigación en Infectología e Inmunología Veterinaria—CI3V, Cra. 30 # 45-03, Bogotá 111321, Colombia; dsvargasb@unal.edu.co (D.S.V.-B.); josedmogollon@yahoo.es (J.D.M.)

**Keywords:** PCV2, PCV3, PPV1, PRRSV, novel parvoviruses (nPPVs), coinfections, porcine reproductive failure (PRF)

## Abstract

Porcine reproductive failure (PRF) is a complex that affects reproductive parameters, leading to significant economic losses for intensive swine farms worldwide. The causes of PRF involve multiple infectious agents, classified into two main groups: primary or putative viruses, which include PCV2, PPV1, and PRRSV, and secondary or occasional viruses, such as PCV3, PCV4, and the new parvoviruses (nPPVs, PPV2 through PPV8). This review provides an updated overview of both viral groups, detailing their unique characteristics and the most commonly reported clinical signs and lesions linked to the putative viruses. While the impact of primary viruses on PRF is well established, the role of secondary viruses in PRF is still under investigation. PCV3 has been directly associated with PRF, characterized by proposed histopathological lesions. Although PCV4 has been identified in reproductive samples, its role in PRF remains unclear. Additionally, nPPVs have been found in reproductive tissues; however, a clear causal relationship with PRF has not been established. The sporadic presence of nPPVs raises questions about their direct impact on PRF and whether they may have synergistic effects when combined with other viruses. This review highlights the growing importance of viral coinfections in the context of PRF. To date, the most frequently reported coinfections are PCV2/PRRSV and PCV2/PPV1, along with emerging pairings such as PCV2/PCV3 and combinations of these two PCVs with nPPVs. Based on the existing literature and our recent findings, we propose a subclinical presentation of PRF, characterized by the presence of both primary and secondary viruses in asymptomatic sows with low viral loads. Furthermore, the synergistic effects of these viruses could contribute to a clinical form of the disease.

## 1. Introduction

Porcine reproductive failure (PRF) is a complex syndrome that significantly impacts swine production worldwide, resulting in substantial economic losses. This syndrome affects various reproductive parameters, leading to decreased conception rates, irregular returns to estrus, increased abortion rates, and a higher number of weak piglets, stillbirths, and mummified fetuses [1]. The causes of PRF can be classified into both non-infectious and infectious categories. Non-infectious factors account for approximately 70% of cases and are mainly linked to environmental conditions, nutritional deficiencies, exposure to toxins, and stress [2]. Infectious causes can stem from the systemic effects of maternal diseases or form direct infections affecting the fetoplacental unit [2]. In recent decades, several viral infectious agents associated with PRF have been identified and categorized into two groups: putative or primary (those with established causality) and associated agents (those lacking definitive proof of causality). Among the primary agents, three main viruses have been identified: porcine circovirus type 2 (PCV2), porcine parvovirus 1 (PPV1), and porcine reproductive and respiratory syndrome virus (PRRSV) [3,4,5]. The associated viruses include certain ones that must be reported to the World Organization for Animal Health (WOAH), such as the classical swine fever virus (CSFV) [6] and the pseudorabies virus (PRV) [7]. Additionally, various other viruses have been sporadically linked to PRF, including porcine enterovirus [8], encephalomyocarditis virus (EMCV) [9], swine influenza virus (SIV) [10], bovine viral diarrhea virus (BVDV) [11], porcine epidemic diarrhea virus (PEDV) [12], and African swine fever virus (ASFV) [13]. Recent advancements in sequencing methodologies and metagenomic analysis have enabled the identification of emerging viruses found in serum and reproductive samples from sows exhibiting signs of SMEDI (stillbirth, mummified fetuses, embryonic death, and infertility). These emerging viruses include porcine circovirus type 3 (PCV3) [14], porcine circovirus type 4 (PCV4) [15], novel porcine parvoviruses (nPPVs, encompassing PPV2 through PPV7) [16], and porcine morbillivirus [17]. The role and implications of emerging viruses in the pathogenesis of PRF are still under investigation and require further exploration. Conversely, several bacterial pathogens have been associated with PRF. These include *Chlamydia* spp. [18], *Erysipelothrix* spp. [19], *Leptospira* spp. [20], and *Brucella suis* [21]. It is important to note that *Brucella suis* is subject to official control and notification requirements in many countries [22]. Furthermore, certain bacteria are classified as opportunistic pathogens, such as *Streptococcus* spp., *Staphylococcus* spp., and *Escherichia coli* [23]. These bacteria can enter the uterus through retrograde transmission via an open cervix or through the bloodstream (bacteremia) [24]. However, this review does not focus on bacterial agents.

The understanding of PRF is evolving due to the emergence of new viruses and the increasing detection of simultaneous viral infections, commonly referred to as coinfections. The most documented viral coinfections associated with SMEDI include PCV2/PRRSV [25,26] and PCV2/PPV1 [20,27]. Recent studies have expanded our understanding of these coinfections also to include PCV2/PCV3 [20], as well as the concurrent presence of these two PCVs alongside nPPVs [16,28]. However, the implications of such coinfections are still not fully understood. There is a lack of objective parameters to differentiate various aspects, such as the pathological effects (both macroscopic and microscopic lesions), the impact on fetal and maternal tissues, and the clinical signs they induce. These findings underscore the complexity of viral infections associated with PRF and highlight the need for a comprehensive approach to diagnose and manage PRF in swine herds accurately. With this context in mind, this review aims to provide a comprehensive and up-to-date discussion on the association between SMEDI and viral causes. It will address primary viruses, including PCV2, PPV1, and PRRSV, as well as emerging viruses, including PCV3, PCV4, and nPPVs. Additionally, the review will explore the coinfections observed among these pathogens. The objective of this work is to evaluate the current understanding of viral infections and coinfections linked to PRF. A thorough literature search was conducted using the PubMed, Web of Science, and Scopus databases, covering research published from 1982 to 2025. A combination of keywords was utilized, including “porcine reproductive failure”, “PCV3”, “PCV4”, “novel porcine parvoviruses”, “viral coinfections”, and “SMEDI syndrome”. Global Studies were included if they reported on PRF, viral detection in reproductive tissues, experimental infections, or epidemiological associations related to PRF in gilts and sows.

## 2. Structural Characteristics of PCVs, PPVs, and PRRSV

### 2.1. Porcine Circovirus

**Figure 1:**
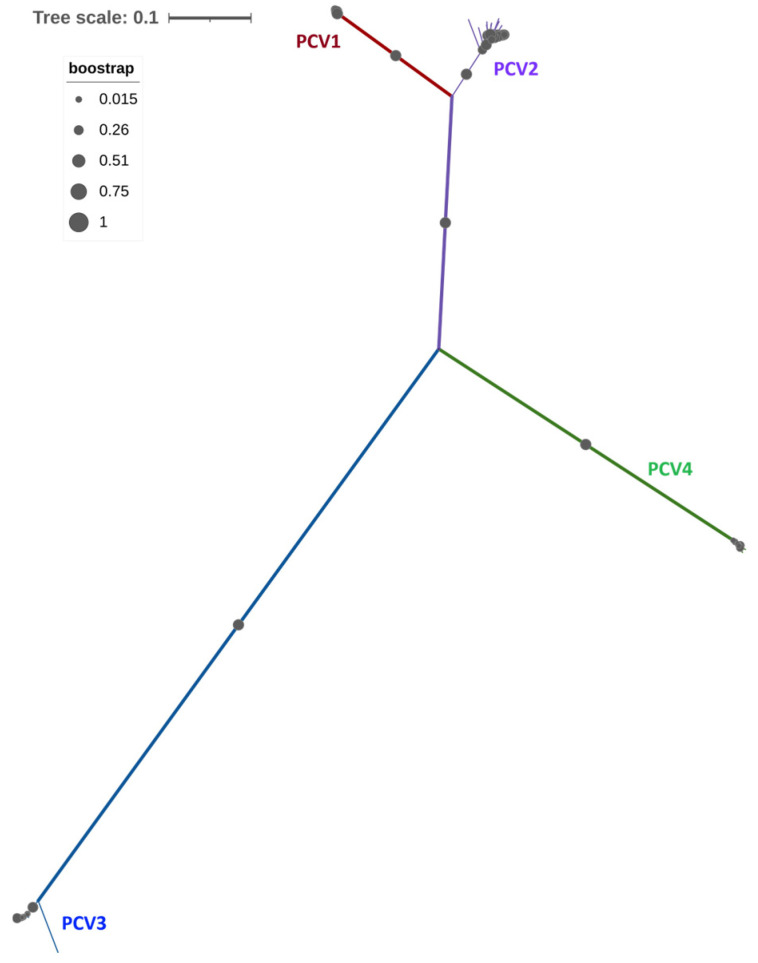
Phylogenetic distribution of porcine circoviruses (PCVs) based on the alignment of 50 representative whole-genome nucleotide (nt) sequences from PCV1 to PCV4, retrieved from the NCBI Genbank nt database. The phylogenetic tree was constructed using the Neighbor-joining method and the p-distance model. The analysis was performed using MEGA-version 11.0 [49]. Bootstrap values are indicated by grey circles. PCV1 genotypes are indicated in red, PCV2 in purple, PCV3 in blue, and PCV4 in green.

PCVs are part of the *Circoviridae* family and are classified under the genus *Circovirus*. Four distinct species have been identified: PCV1, PCV2, PCV3, and PCV4 [29]. Structurally, PCVs are non-enveloped viruses that exhibit icosahedral symmetry, with an average diameter of 25 nm [30]. The viral genome consists of circular single-stranded DNA (cssDNA) that ranges in size from 1.8 to 2.0 kb, with variations observed among the different PCVs species [31,32,33]. The PCV-DNA consists of two main open reading frames (ORFs): ORF1 and ORF2 [34]. ORF1 encodes Rep proteins, which are essential for viral replication [35], while ORF2 encodes the capsid protein (Cap), the only structural component and the most immunogenic protein of the virus [34]. Historically, PCV1 was first identified in the 1970s as a contaminant in the PK15 cell line and is considered non-pathogenic in swine populations, with no clinical signs reported for either natural or experimental infections [36]. In contrast, PCV2 was identified in Canada during the 1990s in pigs that exhibited clinical signs associated with postweaning multisystemic wasting syndrome (PMWS) [37]. PCV2 is currently endemic in swine populations worldwide and is considered the primary causative agent of a group of diseases collectively known as porcine circovirus-associated diseases (PCVAD) [38]. Nine distinct genotypes of PCV2, labeled from PCV2a to PCV2i, have been identified based on variations in the nucleotide (nt) sequence of the ORF2 gene [39,40]. Among these, PCV2a, PCV2b, and PCV2d are widespread globally, while the other genotypes are found sporadically and tend to be geographically restricted. PCV3 was first identified in the USA in 2016, associated with myocardial lesions, porcine dermatitis nephropathy syndrome (PDNS), and sows suffering from PRF [32]. It is important to note that this initial identification was based on associative evidence gathered from field cases. PCV3 is classified into two genotypes, PCV3a and PCV3b, based on nt modifications in the ORF2 and across the entire genome [41]. In 2019, PCV4 was first identified in China in both asymptomatic and symptomatic pigs, the latter exhibiting respiratory symptoms and enteritis [42]. After its initial identification, PCV4 was subsequently found outside of China, including in South Korea [43] and Thailand [44], and then in Europe in late 2023 [45]. Most recently, it was detected in North America in 2024 from samples collected from nursery and fattening pigs affected by enteric and respiratory diseases [46]. PCV4 is classified into two genotypes, PCV4a and PCV4b, based on whole genome analyses and studies of the Rep and Cap genes [47]. A phylogenetic comparison among the four types of PCVs (PCV1, PCV2, PCV3, and PCV4) reveals a higher nt sequence identity between PCV1 and PCV2, ranging from 68% to 76%. In contrast, the identity between these two and the other PCVs (PCV3 and PCV4) is less than 50% [48]. This suggests that there is a limited cross-immune response between these two groups of PCVs. Figure 1 and Figure S1 illustrate the genetic distances among different strains of PCVs. The association between PRF and the phylogenetic classification of PCV2 is primarily evident in genotypes PCV2a, PCV2b, and PCV2d. These specific genotypes are associated with transplacental transmission and have been linked to fetal disease [4,26]. In contrast, other genotypes, such as PCV2c and PCV2e through PCV2i, are reported less frequently, making it difficult to determine whether they have clear connections to PRF.

The associations between the phylogenetic distribution of PCV3 strains and the presentation of PRF have shown limited results. To date, only one study [50] has identified specific associations, revealing that most PCV3 sequences linked to the clinical presentation of PRF belonged to the PCV3c clade. Additionally, this study identified one sequence from the PCV3b clade and one from the PCV3a clade in a piglet that was infected perinatally. It is important to note that the classification of PCV3 used in this study was the one established before 2020, prior to the implementation of the current classification [41]. This limited evidence indicated a possible connection between specific clades and reproductive pathogenicity; however, further investigation is necessary. In our current phylogenetic analyses, we utilized a diverse collection of worldwide sequences categorized into different clades. This included sequences of PCV3 associated with clinical presentations, as well as those from asymptomatic animals that did not exhibit clinical signs. Our findings showed that all clades were linked to the presence of PRF, regardless of whether the sequences were associated with clinical signs or not. Both groups exhibited close phylogenetic relationships, indicating that reproductive pathogenicity is not confined to specific clades. Furthermore, we did not observe any geographic clustering of clades associated with PRF presence. A phylogenetic analysis similar to that conducted for PCV3 could not be performed for PCV4 due to the limited number of sequences available in the databases, especially those from regions outside Asia. Furthermore, no sequences of PCV4 linked to reproductive cases have been reported to date, which complicates the analysis of the specific role of PCV4 in PRF.

### 2.2. Porcine Parvoviruses

Porcine parvoviruses (PPVs) belong to the family *Parvoviridae.* As of now, eight species of PPVs have been identified, with PPV1 being the oldest and most thoroughly studied. The species from PPV2 to PPV8 are collectively known as novel PPVs (nPPVs) [51]. Of these eight species, seven are classified under the subfamily *Parvovirinae* (PPV1 to PPV6 and PPV8), while PPV7 is categorized within the subfamily *Hamaparvovirinae* [52]. The genome of all PPVs consists of linear single-stranded DNA (ssDNA), with an average length ranging from 4 to 6 kb, which can vary depending on the specific PPV species. The ssDNA structure features palindromic sequences at both the 5′ and 3′ ends, resulting in hairpin structures that range from 120 to 200 nt in length. Moreover, PPV-DNA contains two main ORFs: ORF1 and ORF2 [53]. ORF1 encodes the replication-associated proteins (NS or Rep) [54], while ORF2 encodes the structural proteins (VP or Cap), which form the viral capsid [55]. PPVs exhibit significant genetic diversity, primarily due to mutation and recombination events. Recent evolutionary studies have confirmed the virus’s significant capacity for genetic changes, particularly in ORF2, which has an estimated mutation rate of 10^−4^ to 10^−5^ nucleotide substitutions per year (nspy) [56].

**Figure 2:**
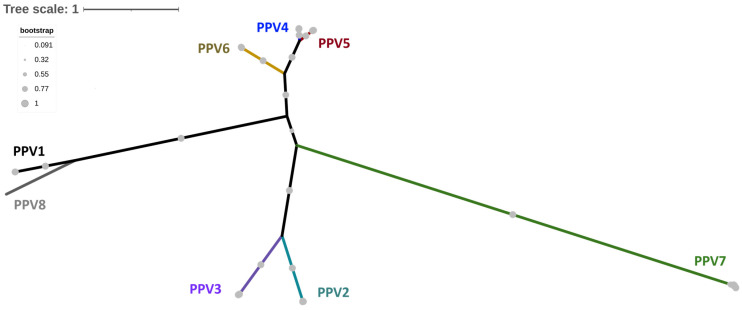
Phylogenetic distribution of porcine parvoviruses (PPV1 through PPV8). This distribution was determined by aligning 200 representatives nonstructural (NS) gene sequences retrieved from the NCBI GenBank nucleotide database. The phylogenetic tree was constructed using the Neighbor-joining method with the p-distance model. Bootstrap values are indicated by grey circles. The analysis was performed using MEGA-version 11.0 [49]. PPV1 strains are represented in black, PPV2 in light blue, PPV3 in purple, PPV4 in dark blue, PPV5 in red, PPV6 in yellow, and PPV7 in green.

The following section provides a clear description of each type of PPV. PPV1 was first identified in the 1960s and has been phylogenetically classified in various ways over the past fifty years. Currently, its classification relies on the variability of the VP protein, which is divided into two main clades: PPV1-I and PPV1-II, each of which contains its subclades [57]. As for the nPPVs, PPV2 was initially identified in Myanmar in 2001 from porcine serum samples [58]. Since its discovery, it has been detected on all continents [59,60,61], indicating a global distribution and rapid spread. Phylogenetic analysis has categorized PPV2 into two main clades, PPV2-I and PPV2-II, with four subclades within clade II [57]. PPV3 was first identified in Hong Kong in 2008 from abattoir samples [62] and has since been detected across Europe [63], Asia [64], the Americas [65], and Africa [66]. Our phylogenetic classification suggests that PPV3 consists of two main clades (PPV3-I and PPV3-II) and five subclades within clade II [57]. Moving to PPV4, it was first identified in the USA in 2010 [67] and since its discovery, it has been found on every continent except Oceania [61,63,68]. Recent phylogenetic analyses suggest that PPV4 can be categorized into three main clades: PPV4-I, PPV4-II, and PPV4-III, with further subclade divisions in both PPV4-II and PPV4-III [57]. PPV5 was initially identified in the USA in 2013, based on lung and stool samples from pigs exhibiting various syndromes [69,70]. Since then, it has been detected in Asia [68,71], Europe [72], and the Americas [28,73]. We proposed categorizing PPV5 into three clades, PPV5-I, PPV5-II, and PPV5-III, with two subclades within PPV5-I and two within PPV5-III [57]. PPV6 was first identified in China in 2014 from samples collected from aborted fetuses, piglets, sows, and gilts in both symptomatic and asymptomatic herds [74]. It has also been detected in North America [75], Europe [72,76], Asia [68], and the Americas [73]. Our proposed phylogenetic classification for PPV6 includes two clades: PPV6-I and PPV6-II. Within PPV6-I, there are two subclades, while PPV6-II consists of three subclades [57]. PPV7 was initially identified in the USA in 2016 through a metagenomic analysis of fecal samples [77] and has since been detected on several continents [78,79] from various sample types [80,81]. Based on phylogenetic analysis, we proposed a classification of PPV7 into two main clades, PPV7-I and PPV7-II, each containing two subclades [57]. PPV8, the latest identified member of the nPPVs, was first detected in lung samples in China in 2022 [82]. It was subsequently reported in Europe in 2024, specifically in Hungary and Slovakia [83], and more recently in the Americas, with notable detections in Colombia in August 2024 [84]. The evolutionary origins of these PPVs (PPV1 through PPV8) remain unclear, but genetic analyses reveal substantial sequence diversity. Comparative genomic studies indicate variations in identity ranging from 33% to 90%, with PPV7 showing the highest level of divergence (Figure 2 and Figure S2). This significant genetic variability among nPPVs highlights the necessity for ongoing surveillance and phylogenetic analysis.

### 2.3. PRRSV

**Figure 3:**
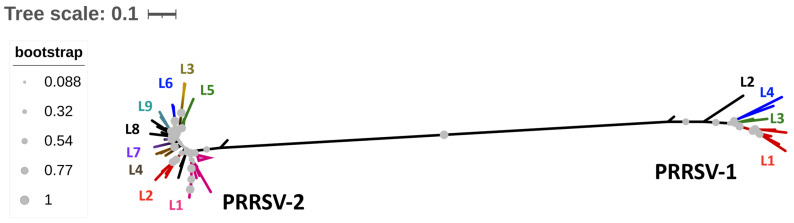
Phylogenetic distribution of porcine reproductive and respiratory syndrome virus (PRRSV). This analysis is based on the alignment of 80 representative ORF5 nucleotide (nt) sequences from PRRSV-1 and PPRSV-2, which are available in the NCBI GenBank nt database. The phylogenetic tree was constructed using the Neighbor-joining method along with the K2 + G + I model. Bootstrap values are indicated by grey circles. The analysis was performed using MEGA-version 11.0 [49]. The PRRSV-1 lineages are depicted on the right side of the graph using different colors: L1 is red, L2 is black, L3 is green, and L4 is blue. The PRRSV-2 lineages are shown in various colors as well: L1 is pink, L2 is red, L3 is yellow, L4 is brown, L5 is green, L6 is blue, L7 is purple, and L8 is black.

The PRRSV belongs to the *Arteriviridae* family and the *Betaarterivirus* genus. Two distinct species of PRRSV have been identified: *Betaarterivirus* suid 1 (PRRSV-1) and *Betaarterivirus* suid 2 (PRRSV-2) [85]. These species are found globally, with PRRSV-1 being more prevalent in Europe and PRRSV-2 being more common in America and Asia. Structurally, PRRSV is an enveloped virus with pleomorphic symmetry, measuring between 50 and 70 nm in diameter [86]. Its genome consists of positive-sense, single-stranded RNA (ssRNA) that is approximately 15 kilobases (kb) in length. This genome consists of nine ORFs: ORF1a, ORF1b, ORF2a, ORF2b, and ORF3 through ORF7 [87]. ORF1a and ORF1b account for two-thirds of the genome and are translated into a polyprotein known as ORF1a/b. This polyprotein is then cleaved into 16 nonstructural proteins (Nsp1 to Nsp16), which are involved in viral replication, genome transcription, and the modulation of host cell immune responses [88]. The remaining ORFs, from ORF2 to ORF7, encode six subgenomic RNAs that produce structural proteins: GP2 to GP5, M, and N. Specifically, ORF2, ORF3, and ORF4 encode the minor envelope proteins GP2, GP3, and GP4, respectively. The major envelope proteins, GP5 and M, are encoded by ORF5 and ORF6, respectively, and they form a heterodimer. Notably, ORF5 exhibits high variability, which is advantageous for phylogenetic analysis and viral classification [89]. ORF7 encodes the nucleocapsid (N) protein [88]. Significant genetic and antigenic diversity has been observed within each PRRSV species [90]. Phylogenetic analyses of ORF5 have classified PRRSV-1 into four subtypes (I to IV) [91]. In contrast, the classification of PRRSV-2 is determined through ORF5 sequencing and restriction fragment length polymorphism (RFLP) methods [92,93], resulting in nine distinct lineages (L1 to L9), along with alphabetically designated sublineages [89]. Currently, the most prevalent PRRSV-2 lineages in the USA are L1, L5, L8, and L9, with sublineages L1A, L1B, and L1C being the most common [94]. Recent research on the evolutionary dynamics of PRRSV-2 has uncovered the emergence of variants within these sublineages [95]. RFLP analyses of PRRSV-2-ORF5 patterns, using three restriction enzymes (*MlluI*, *HincII*, and *SacI*), have been employed to classify and infer potential pathogenic biotypes. However, the genetic relationship between different RFLP types remains unclear; phylogenetically distant viruses may share the same RFLP pattern, while viruses from the same lineage can exhibit different patterns [96]. Figure 3 and Figure S3 illustrate the genetic distances between various PRRSV species and their lineages. The extensive diversity observed among PRRSV strains can be attributed to the low fidelity of the viral RNA polymerase (RdRp), which leads to two main consequences: (i) the introduction of mutations in newly synthesized viral RNA copies [97] and (ii) high recombination rates between different PRRSV viral genomes [98]. These factors contribute to the periodic emergence of novel PRRSV strains with altered virulence [99].

Both PRRSV-1 and PRRSV-2 species are known to cause reproductive disease through transplacental transmission and fetal infection. Comparative studies have not shown significant differences in the severity of PRF between these two species [100]; however, at the intraspecies level, there is variation in virulence among different PRRSV lineages and strains, even though they share the ability to cross the placenta [101]. During the 1990s, the most prevalent strains, particularly the L5 strains (e.g., VR2385 and VR2332), were associated with severe reproductive effects [102,103]. Similarly, strains from lineages L8 and L9 have also been linked to reproductive issues [102,104]. More recently, lineage 1 strains (1-7-4) have been connected to outbreaks of reproductive disease, highlighting the ongoing evolution of reproductive pathogenicity across different lineages [105]. There is limited literature regarding lineages L2, L3, L4, L6, and L7 and their associations with PRF, indicating a gap in knowledge in this area. Furthermore, there are currently insufficient reports documenting which specific PRRSV strains or lineages are most commonly found in coinfection scenarios, which represents an important area for future research.

## 3. Porcine Reproductive Failure (PRF) Can Be Linked to Various Viruses, Including PCVs, PPVs, and PRRSV

Two important aspects of the viruses associated with SMEDI deserve special attention. First, maternal viremia is critical for viral replication in the endometrium, which facilitates fetal transmission through the placenta [106,107]. Second, the clinical signs of SMEDI can vary widely within breeding herds, influenced by factors such as the gestational stage, the virulence of the circulating viral strains, and the immunocompetence of both the sow and the fetuses [20]. The effects of viral infections on porcine pregnancy exhibit distinct patterns depending on the stage of fetal development. During early gestation (0 to 15 days of pregnancy), which coincides with implantation and embryogenesis, the zona pellucida of the zygote acts as a protective barrier against pathogen entry. However, some viruses have evolved mechanisms that enable them to adhere to or penetrate this barrier, potentially leading to embryonic reabsorption and negatively impacting reproductive parameters, such as the litter size [107,108]. The second stage of pregnancy (mild), which occurs at approximately 35 to 70 days, is particularly vulnerable. Viral infections during early pregnancy often lead to fetal mortality, as indicated by an increase in mummified fetuses [109,110,111]. In contrast, diseases that occur during late pregnancy (between 70 and 114 days) present a different scenario. At this stage, fetuses have developed immunocompetence, which theoretically enables them to mount an acquired immune response capable of controlling the infection. However, while viral infections during this period can still negatively impact fetal development, they do not always significantly reduce survival rates. In some cases, infections may result in severe outcomes such as fetal mummification, fetal death, or the birth of weak or healthy piglets [107,110]. Overall, the complex interactions between viral pathogenesis and fetal development highlight the intricate nature of SMEDI.

### 3.1. PRF Associated with Porcine Circovirus Infection

PCV2-associated PRF was first reported in 1999 in gilts that displayed clinical signs such as late-term abortions, pseudopregnancy, high rates of mummified fetuses, autolyzed fetuses, and stillborn piglets [4,112]. Further investigations indicated that pathologies caused by PCV2 can occur in pregnancy, resulting in mummified fetuses of varying crown–rump lengths (CRL) and a higher incidence of stillborns at delivery [25,113,114] (Table 1). To understand the impact of PCV2 on pregnancy, it is important to recognize that PCV2 infection in pregnant sows leads to prolonged viremia [115]. This prolonged presence of the virus in the bloodstream increases the likelihood of in utero transmission between fetuses and contributes to various reproductive anomalies. Furthermore, evidence of intrauterine infection has been documented through the detection of PCV2-DNA, viral antigens, and anti-PCV2 antibodies (Abs) in stillborn piglets, fetuses, and clinically healthy littermates [116,117,118]. Additionally, piglets that survive an in utero infection with PCV2 are known to have an increased susceptibility to PMWS, resulting in higher morbidity and mortality rates [119]. There is significant variability in infection rates among sows; some remain asymptomatic [120], while others display systemic disease symptoms such as pneumonia, diarrhea, wasting, and necrotizing dermatitis, particularly in gilts during the acclimation phase [121]. Despite this variation, it is generally accepted that the incidence of SMEDI associated with PCV2 is relatively low. This is attributed mainly to the high seroprevalence in global swine herds, which is a result of either natural exposure or vaccination programs [122]. In contrast, herds experiencing PCV2-reproductive disease (PCV2-RD) are often either seronegative for PCV2 or have many gilts with incomplete immunity to the virus [123]. These herds can be divided into two categories based on the prevalence of PCV2-DNA: (i) stable herds, which exhibit low or no levels of PCV2-DNA, and (ii) unstable herds, where the prevalence of PCV2-DNA is high (≥50%). It is important to note that this categorization is independent of the presence of anti-PCV2 Abs. Neutralizing Abs are present in both gilts and sows across all herds, and these Abs are passed to piglets as maternally derived antibodies (MDA) during lactation, providing some protection against PCV2 infection [124]. However, the simultaneous presence of both PCV2-DNA and anti-PCV2 Abs in colostrum complicates efforts to control the virus. As a result, piglets may either be born infected or become infected during the lactation phase. The infection dynamics of PCV2 are among its most effective adaptations for persistence within swine populations [125]. The diagnosis of PCV2-RD is confirmed when the number of viral genomic copies exceeds 10^5^, especially in cases involving mummified fetuses and stillbirths [126]. Despite our comprehensive understanding of PCV2’s impact on PRF, many questions remain unanswered, particularly regarding the effects of subclinical PCV2 infections on PRF [127].

The association between PCV3 and PRF was first noted in 2017, linking the virus to increased mortality rates in sows and decreased pregnancy rates [32]. However, it is essential to understand that this relationship is based on data associations rather than definitive causal evidence. Subsequent studies conducted in various countries have detected PCV3-DNA in mummified fetuses and stillborn pigs from both gilts and sows, with detection rates ranging from 1.9% to 100% [129,130,135,136] (Table 2). Regarding the clinical signs associated with PCV3 infection, these were primarily observed during the middle-to-late stages of pregnancy (Table 1). The clinical signs were characterized by an increase in mummified fetuses and stillborn piglets at delivery [137]. It is important to clarify that the clinical presentation of PCV3 can vary across different field studies. Some herds that test positive for PCV3-DNA remain asymptomatic in both gilts and sows [138], suggesting that the mere detection of PCV3 does not necessarily imply causality in every case. In contrast, other herds report higher mortality rates among sows [139,140], along with clinical signs in gilts such as anorexia, lethargy, dyspnea, hypothermia, and PRF [141]. There is compelling evidence linking PCV3 to PRF, as studies show that sows experiencing PRF have higher rates of PCV3-DNA detection (*p* < 0.05) and elevated Abs levels (*p* < 0.01) compared to asymptomatic animals [139,142]. One Spanish study suggested that PCV3 might function as a putative reproductive disease virus (PCV3-RD) due to the correlation observed between PCV3-DNA detection and signs of SMEDI. This association is further supported by observations of late-term abortions, mummified fetuses, stillbirths, piglets, and weak piglets, along with high viral loads of PCV3 in fetal tissues and the presence of related histopathological lesions [143]. The most substantial evidence supporting the causal role of PCV3 in PRF comes from experimental challenge studies. One such study involved pregnant sows infected with PCV3 [144], providing direct causal evidence under controlled conditions. The findings revealed (i) the presence of mummified fetuses and stillborn piglets, (ii) the persistence of PCV3-DNA in the same sow for at least 80 days, and (iii) the detection of PCV3-DNA and systemic lesions in the offspring. This experimental evidence establishes causality rather than merely suggesting an association. Additionally, the presence of anti-PCV3 Abs in fetal tissues indicates that the virus can be transmitted in utero [118]. Other studies have also detected PCV3 in colostrum [145] and semen [146], suggesting that these bodily fluids could be potential sources of viral transmission. Regarding the transfer of maternal Abs, piglets born to PVC3-positive sows show measurable levels of PCV3 Abs that gradually decline, typically becoming undetectable by 6 to 8 weeks after farrowing [147]. The evidence indicates a moderate-to-strong causal relationship between PCV3 and PRF, supported by experimental challenge studies that demonstrate the reproductive pathology.

The relationship between PCV4 and FRP is not yet fully understood due to the limited research available on this topic. Current evidence suggests an associative link rather than a causal one. Reports indicate that PCV4 has been detected in 7.69% of sow serum samples and 4.7% of aborted fetuses [15,42], raising the possibility of vertical transmission [158]. However, these detection rates are based on cross-sectional surveys that do not include groups of healthy reproductive sows. PCV4-specific Abs have been found in pig serum, with the highest prevalence of 67.8% observed in sows [159]. Additionally, anti-PCV4 Abs were identified in 15% of suckling piglets, indicating the transfer of MDA specific to PCV4 [159]. Overall, the evidence supporting a causal relationship between PCV4 and PRF remains insufficient or weak. This is mainly due to the lack of reproductive experimental challenge studies, limited field data without appropriate controls, and the recent discovery of the virus, which restricts the availability of long-term epidemiological data.

### 3.2. PRF Associated with Porcine Parvoviruses Infection

V1 is recognized as the primary viral agent associated with PRF [3]. However, its prevalence has remained low over the past three decades, primarily due to routine herd vaccination programs and effective MDA transfer [160]. These Abs are mainly found in colostrum [161] and gradually decline as piglets grow older. They are typically detectable for up to 12 weeks [162], although some studies have indicated that they can persist for as long as 21 weeks [163]. The impact of PPV1 on FRP is closely related to the stage of gestation at which the infection occurs and the virulence of the viral strain involved (Table 1). During early pregnancy, infection with PPV1 can lead to a return to estrus, resorption, fetal death, and a reduced litter size [107,161]. In mid-pregnancy, the virus can cause fetal death and mummification [109]. In contrast, infections during late pregnancy usually result in fetal survival, as the fetuses have developed some level of immunocompetence against the virus [163,164]. Nonetheless, some studies have reported occurrences of mummified fetuses and stillbirths during late gestation, particularly when sows are infected with highly pathogenic strains of PPV1 [109]. Since the early 2000s, Europe has reported the circulation of strains known as “divergent PPV1” (PPV1-27a). These strains appear to have a more significant reproductive impact, resulting in higher fetal mortality rates compared to classical strains [164,165]. Clinical signs of PPV1 infection can be minimal or absent, particularly in non-pregnant gilts, sows, and boars [107]. The virus is primarily observed in susceptible, unvaccinated gilts or those with low levels of MDA [166]. Additionally, some reports indicate that intrauterine viral transmission can occur from deceased fetuses to their littermates, with infections manifesting later in the gestation period during mid-to-late pregnancy [107]. In boars, PPV1 has been isolated from semen, which poses a potential risk of venereal transmission [166].

Recent research has explored the potential link between nPPVs and PRF. However, the current evidence is primarily observational and lacks definitive causal validation. Various viral species, specifically PPV2 through PPV7, have been detected in aborted fetuses, with prevalence rates ranging from 2.4% to 96% [16,28] (Table 2). Additionally, these nPPVs have also been found in the serum of gilts without PRF, which challenge their pathogenic role in PRF [26,153]. This wide variation in prevalence rates suggests the influence of significant confounding factors, differing diagnostic methodologies, population heterogeneity, or varying sample types, which limits the strength of the associative conclusions. Among these species, PPV2 is considered the most likely candidate associated with SMEDI syndrome [167]. This assessment is mainly on associative evidence from field studies rather than experimental validation, supported by variable detection rates ranging from 2.4% to 96% in aborted fetuses [16,28], along with its presence in 26.3% of stillborn piglets [155]. Overall, the current evidence supporting a causal association between PPV2 and PRF remains weak or insufficient. Findings on the other nPPVs are at preliminary stages, with evidence limited to associative data from cross-sectional surveys that lack appropriate control groups from healthy reproductive herds or tissues. Notably, PPV4, PPV6, and PPV7 have been detected in herds experiencing PRF, with detection rates reaching up to 50% in aborted fetuses [60,74,152]. Notably, PPV4 is the only nPPV detected in ovarian and uterine tissues [168], indicating a potential affinity for reproductive organs. PPV3 and PPV5 have also been identified in aborted fetuses, with prevalence rates ranging from 3.2% to 38% for PPV3 and from 18.8% to 28% for PPV5 [16,28,157] (Table 2). The current evidence for nPPVs has notable limitations that hinder causal inference in PRF. The existing detection studies offer only descriptive data without control comparisons, lack in situ or immunohistochemical confirmations in fetal tissues, and are based on uncontrolled field observations. Furthermore, the absence of nPPVs in colostrum and milk samples suggests limited lactational transmission; however, this finding is derived from small sample sizes and needs validation across diverse populations. To date, PPV8 has not been detected in any reproductive samples.

### 3.3. PRF Associated with PRRSV Infection

PRRSV is a reproductive pathogen that causes a range of clinical conditions, which can vary based on the pregnancy stage, the immune status of the sows, and the specific viral strain in circulation [5]. The most common clinical signs of PRRSV are generally observed during late pregnancy and include abortions, premature farrowing (occurring at 107 to 112 days of gestation), an increase in stillborn piglets, mummified fetuses, and weak-born piglets at birth [111]. While less frequently reported, some studies have also noted the effects of PRRSV during early gestation [132,133] (Table 1). The exact mechanism by which PRRSV crosses the maternal–fetal interface is not fully understood. However, it is known that macrophages expressing CD163 on their surface are the primary target for the virus during pregnancy [169]. Notably, during early and mid-gestation, the predominant macrophage phenotype is CD163+Sn-, which shows relative resistance to viral infection. In contrast, late pregnancy is characterized by the presence of CD163+Sn+ macrophages, indicating an increased susceptibility to infection [169]. The severity of PRRSV infection can vary significantly among littermates that share the same uterine environment [170]. This variability leads to a range of outcomes, including asymptomatic piglets, weak-born piglets, decomposed or autolyzed fetuses, stillborns, and occasionally, meconium-stained fetuses [171,172]. Asymptomatic piglets that survive an intrauterine infection may continue to replicate the virus persistently within their lymphoid tissue for up to 132 to 210 days of age [173]. Recent studies have proposed three categories to help explain the differences in fetal responses to PRRSV infection: (i) fetal susceptibility—this category is characterized by high viral loads found in multiple fetal tissues, which are often associated with non-viable fetuses, such as meconium-stained stillborns or decomposed fetuses [170]—(ii) fetal resilience—this classification includes viable fetuses that display high PRRSV viral loads across various tissues, but remain clinically asymptomatic [174]—and (iii) fetal resistance—this category refers to some fetuses that can control viral replication, resulting in asymptomatic piglets with low or undetectable viral loads in most fetal tissues.

## 4. Fetal Lesions Associated with Infection Caused by PCVs, PPVs, and PRRSV

Reproductive disorders caused by viral infections primarily arise from the infection of the fetoplacental unit. Diagnosing viral infections in fetuses involves detecting the virus and analyzing both macroscopic and microscopic lesions. A significant challenge is that the macroscopic appearance of mummified fetuses infected by the viruses discussed in this review is quite similar, making it difficult to establish the specific causality [175]. Additionally, a histopathological examination of fetal tissue often reveals infrequent or nonspecific microscopic lesions [150,176,177]. In the case of PCV2, the lesions observed in fetuses vary depending on the pregnancy stage at which the infection occurs. In fetuses aborted during mid-gestation, microscopic lesions are found in the myocardium, which may include degeneration, necrosis, nonsuppurative myocarditis, fibrosis, and mineralization. In later stages of pregnancy, lesions primarily affect the lymphoid organs of fetuses and neonates, with lymphoid depletion being the most frequently reported issue (Table 3) [178]. For PCV3, the most commonly observed lesions are located in the myocardium and nervous tissue, especially during early or mid-gestation [144]. Several studies have investigated the impacts of both PCV2 and PCV3 on fetuses and found that, even with high viral loads, establishing a clear link to histopathological lesions remains difficult [114,141]. Lesions related to PCV3 are more often seen in stillborn and weak-born piglets and are marked by multisystemic lymphoplasmacytic to lymphohistiocytic perivascular inflammation [143]. Other lesions in piglets may include lymphohistiocytic myocarditis, interstitial pneumonia, nonsuppurative encephalitis, and interstitial nephritis [50] (Table 3).

In infections caused by PPV1, lesions have been observed in both maternal and fetal tissues. In sows, the primary site of lesions is the placenta, which can exhibit conditions such as placentitis, calcification, diffuse hemorrhages, and edema [176,180]. Identifying specific lesions in fetuses with CRL < 17 cm is challenging due to autolysis. However, in fetuses with a CRL > 17 cm, the lesions include the perivascular infiltration of mononuclear cells in various organs, the necrosis of cells in developing organ systems, and mineralization in the lungs, kidneys, liver, and heart [3,182]. Current evidence suggests that fetal lesions are more severe and more frequently observed in infections caused by “PPV1-divergent” strains (Table 3) [180]. Lesions associated with PRRSV have primarily been found in the tissues of sows. These include uterine edema, lymphohistiocytic endometritis, lymphocytic myometritis, and lymphoplasmacytic and histiocytic placentitis [5,177,183]. Additionally, lesions have been observed in newborn piglets, affecting various organs such as the umbilical cords (which may exhibit hemorrhaging and edema), heart, mesenteric lymph nodes, lungs, and the central nervous system (Table 3). Microscopic lesions are more frequently seen in weak-born piglets compared to fetuses or stillborns [183], commonly manifesting as interstitial pneumonia, nonsuppurative myocarditis, and nonsuppurative encephalitis [172].

## 5. Proposed Subclinical Presentation of Porcine Reproductive Failure Caused by PCV2, PPV1, and PRRSV

**Figure 4:**
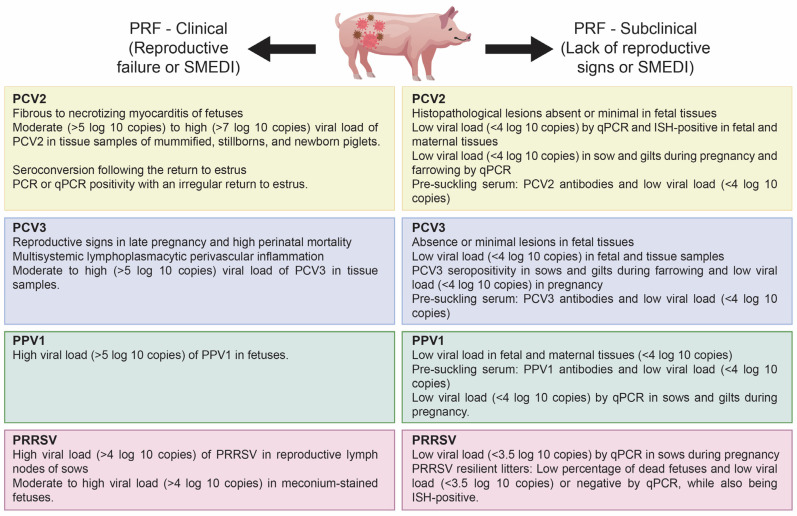
Clinical signs and diagnostic profiles of the primary viruses (PCV2, PPV1, and PRRSV) responsible for porcine reproductive failure identified in the clinical form (PRF-C) and those suggested for the subclinical form (PRF-SC). This diagram was created using Illustrator version CS5.

The clinical signs and reproductive effects associated with primary PRF viruses, specifically PCV2, PPV1, and PRRSV, have been well-documented in the existing literature. Although these viruses are commonly found in pig populations, there has been a notable decrease in the frequency of clinical symptoms. This observation suggests that the viruses may persist subclinically, particularly within reproductive tissues. Such a situation presents new challenges for detection, diagnosis, and control strategies. Currently, the criteria for identifying subclinical infections related to PRF are limited. Based on previously published reports and our findings, we propose classifying PRF into two categories: (i) clinical porcine reproductive failure (PRF-C) and (ii) subclinical porcine reproductive failure (PRF-SC). This classification is based on the detection of the suspected PRF viruses (PCV2, PPV1, and PRRSV) in sows experiencing subclinical infection during pregnancy, farrowing, and lactation phases [26] (Figure 4).

The epidemiology of PCV2, PPV1, and PRRSV in pig populations has undergone significant changes in recent years. This shift is primarily due to the widespread presence of these viruses in pig herds and the implementation of extensive vaccination programs aimed at controlling them. These factors have added complexities to the interpretation of serological results in sows and their offspring. For example, while serum viremia observed before suckling indicates fetal infection, it does not provide definitive evidence regarding the relationship between these viruses and PRF. Widespread and continuous vaccination protocols for PCV2 in gilts and piglets have resulted in a significant shift from clinical to subclinical disease presentations in most herds [184]. The evidence suggests that subclinical PCV2 infections are associated with incomplete vaccine efficacy, resulting in non-sterilizing immunity [185]. This subclinical form, referred to as PCV2-subclinical infection (PCV2-SI), is characterized by the absence of clinical signs (asymptomatic pigs), minimal or no histopathological lesions, and low viral loads in tissues [38]. Similarly, PRRSV infections in endemic herds often display mild or no signs (subclinical), with minimal effects on reproductive parameters [186]. While PPV1 is actively involved in causing PRF, it is also recognized as a factor contributing to mild or subclinical infections [63]. Mass vaccination against PPV1 may alter its epidemiology, leading to an increase in predominant subclinical infections, a trend similar to what has been observed with PCV2. Since its initial identification, PCV3 has been detected in sows globally, often showing no clinical signs [26,145,187]. This widespread occurrence suggests that subclinical infection is likely the most common manifestation of PCV3 [143]. Even in the absence of clinical signs, these subclinical infections in sows could have significant underlying effects. Additionally, PCV2 and PRRSV can facilitate coinfections with other pathogens [186,188]. This increasing occurrence of subclinical infections underscores the importance of employing diagnostic methods that focus on their detection.

## 6. Viral Coinfections Linked to Reproductive Failures in Gilts and Sows

A coinfection occurs when two or more pathogens simultaneously infect a cell or host. The effects and clinical significance of coinfections are not fully understood, making this a growing area of research [189]. Field-based diagnostic laboratory surveillance could be essential for accurately quantifying the true prevalence and impact of these coinfections in swine populations worldwide. Current findings from both experimental and field diagnostic data suggest that viral coinfections can lead to various outcomes, including (i) viral interference, where one virus suppresses the replication of another coinfecting virus; (ii) the enhanced replication of one of the coinfecting viruses; (iii) increased severity of the disease; (iv) exchange of genetic material between viruses, resulting in recombinant strains, and (v) coexistence of coinfecting viruses without affecting the replication of any of the viruses [189]. Significantly, the impact of a coinfection is influenced by the host’s immune response, as both humoral and cellular immune memory can shape the quantity and quality of the response to the coinfection [189]. In the context of PRF, various coinfections have been identified in aborted fetuses, stillborn pigs, weak-born piglets, and sows through routine diagnostic submissions to veterinary laboratories worldwide. These infections have been detected in serum samples and tissues from the reproductive organs. Recent studies have employed genomic detection methods, including PCR and RT-PCR, to identify concurrent viral infections. The most commonly detected coinfections include PCV2/PRRSV and PCV2/PPV1. More recently, coinfections involving PCV2/PCV3 have also been documented, along with cases that include these two PCVs and nPPVs.

To assess the prevalence of viral coinfections in cases of PRF, we analyzed data from 22 veterinary diagnostic laboratories located in North America, Asia, Europe, and South America, covering the period from 1982 to 2025. Our comprehensive analyses included samples from aborted fetuses, stillborn piglets, and sera collected from sows experiencing PRF, using PCR as the detection method. This field-based approach provides crucial epidemiological evidence that underscores the clinical significance of viral coinfections in PRF cases. Our global review revealed distinct geographical patterns in the prevalence of coinfections, highlighting significant regional differences in their epidemiology. All continents reported low-to-moderate prevalence rates for the established coinfection: for example, PCV2/PPV1 ranged from 1% to 20%, PRRSV/PPV1 from 1% to 9%, and PCV2/PRRSV from 0.5% to 45%. Notably, Asia exhibited the broadest range of prevalence for PCV2/PRRSV coinfections, suggesting either epidemiological differences in the circulation patterns of the viruses or variations in the detection methods used across different laboratories. Of particular concern are the coinfections involving emerging viruses, which displayed substantially higher rates, ranging from 1% to 98%. This indicates the rapid evolution of interactions among viral pathogens within swine populations. For instance, the prevalence of the PCV2/PPV2 coinfection reached an astonishing 98% in fetal tissues from Mexico, a significant contrast to the lower rates observed in other regions for the same viral combination. This disparity suggests that high viral circulation may be specific to certain areas or related to methodological differences in the sample collection and processing. Overall, the data presented in Table 4 indicate that some coinfections have become endemic in multiple geographical regions, whereas others appear to be specific to certain areas. These varying prevalence rates reflect local epidemiological conditions, management practices, and the diversity of the viruses involved.

The surveillance period from 1982 to 2025 revealed distinct phases in the detection patterns of viral coinfections, reflecting the historical emergence and evolution of swine viral pathogens. From the beginning of this period until 2010, coinfections involving PCV2/PRRSV and PCV2/PPV1 were commonly reported in diagnostic reports. This dominance mirrored the historical emergence and establishment of these viral combinations in global swine populations. A significant epidemiological shift was observed after 2015, marked by an increase in the detection of coinfections associated with PCV3 and nPPVs combinations. This change corresponds with the discovery and increasing recognition of these emerging pathogens. These evolving dynamics of coinfections indicate that they continuously adapt as new viral pathogens emerge and interact with established viruses, creating increasingly complex epidemiological challenges that traditional methods of diagnosis and management may struggle to address.

The widespread detection of multiple viral coinfections across diverse geographical regions, with some combinations showing prevalence rates exceeding 20% in various locations, strongly supports the clinical hypothesis that these coinfections play a crucial role in the pathogenesis of PRF. The epidemiological evidence indicates that single-pathogen approaches for PRF diagnosis may be inadequate to capture the full spectrum of viral involvement in PRF cases. Moreover, the emergence of novel coinfection patterns, particularly those involving new viruses, highlights that the viral landscape affecting swine reproduction is dynamic and continually evolving. The substantial geographical and temporal variations in coinfection prevalence rates carry significant implications for future research. These wide prevalence ranges suggest that local epidemiological factors and variations in detection methodologies greatly influence coinfection dynamics, underscoring the necessity for standardized diagnostic approaches.

Viral coinfections may have an impact on PRF; however, the specific effects, including both macroscopic and microscopic lesions, as well as clinical signs, are not yet fully understood. Table 5 provides a detailed overview of viral coinfections associated with PRF, along with the related lesions. One notable coinfection is PCV2/PPV1. The evidence suggests a potential synergistic effect on PRF, as seen in several observations: (i) fetuses infected with the PCV2/PPV1 coinfection exhibited more severe lesions compared to those infected with only PPV1 or PCV2 [27,195]; (ii) there was a higher incidence of small mummified fetuses (CRL < 17 cm) in cases of PCV2/PPV1 coinfection [20]; and (iii) a significantly greater prevalence of PPV1 infections was observed in PCV2-positive gilts compared to those that were PCV2-negative [153]. Another commonly noted coinfection associated with PRF is PCV2/PPRSV. In this case, the simultaneous replication of both viruses has been detected in the same fetal organ, specifically in the myocardium. However, this concurrent replication did not worsen the severity of the lesions [196].

Coinfections involving PCV3 and other PRF-associated viruses (including PCV2, PPV1, and PRRSV) have been documented. These coinfections correlate with reproductive issues such as abortions, fetal mummification, and stillbirths [198]. The evidence of coinfection during sow pregnancy is supported by the simultaneous detection of anti-PCV2 and anti-PCV3 Abs in fetal tissues. Interestingly, one study observed a reduced likelihood of detecting PCV2 when PCV3 was present in fetal tissues, suggesting that PCV3 may inhibit PCV2 during transplacental infection [20]. Additionally, the significant presence of PCV3 alongside PCV2 raises concerns about its potential impact on the efficacy of vaccines against circoviral infections in breeding sows [118].

Recent investigations have identified coinfections in aborted fetuses involving nPPVs, specifically PPV2 through PPV7, alongside PCV2, PCV3, and PRRSV [155,157]. These studies on coinfections are primarily descriptive and face significant challenges in establishing causal relationships due to the complexity of multiple viral interactions. The most common combinations of coinfections include PCV2/PPV2, PCV2/PPV5, PCV2/PPV6, PCV2/PPV3, PCV3/PPV3, PCV3/PPV6, PRRSV/PPV2, PRRSV/PPV3, and PCV3/PPV7 [16,28]. Among these combinations, only the coinfections of PCV2/PPV5 and PCV2/PPV6 have been positively associated with an increased incidence of abortions [28], providing the strongest statistical evidence for the pathogenic interaction among nPPVs. Additionally, PCR analyses have revealed distinct patterns in nPPV prevalence across different viral infections. Notably, PPV6 was found to be more common in gilts that tested PCV3-positive, while PPV5 was more prevalent in gilts that were PRRSV-positive [153]. Furthermore, among PCV3-positive sows experiencing PRF, a higher positivity rate for PPV7 has been observed [152].

**Figure 5:**
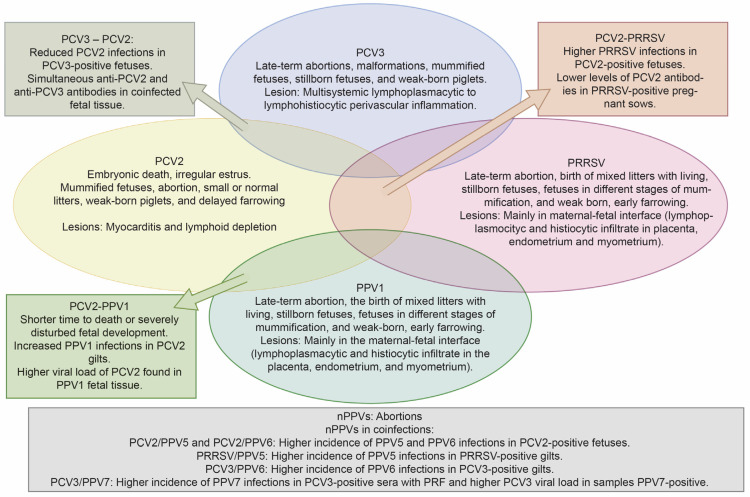
The Venn diagram illustrates the overlapping and distinct reproductive outcomes linked to infections by primary porcine viruses, including PCV2, PPV1, and PRRSV, as well as PCV3. It provides information on both mono- and coinfections, and it also emphasizes the effects that may result from coinfections involving novel porcine parvoviruses (nPPVs). This diagram was created using Illustrator version CS5.

There is growing evidence in the scientific literature that coinfections with both primary and emerging viruses are common in cases of PRF. This issue is complicated by the absence of clear clinical or reproductive patterns associated with viral coinfections in PRF, highlighting the complex interactions among multiple pathogens. The clinical manifestations of mono-infections caused by primary PRF viruses are well-established and distinct. However, this clarity does not apply to coinfections involving these pathogens. In this review, we present a comprehensive overview in Figure 5, highlighting the most commonly observed signs and lesions associated with viral mono-infections of PCV2, PCV3, PPV1, and PRRSV. Additionally, we illustrate the overlapping clinical signs and pathological findings that arise when these primary viruses are involved in coinfections. We also aim to include the effects of coinfections between the primary viruses and nPPVs. It is essential to note that although this figure represents the most frequently reported findings, individual cases may vary due to factors such as the viral strain, host immunity, environmental conditions, and management practices.

## 7. Conclusions

Porcine Reproductive Failure poses one of the most significant challenges in swine production, primarily due to its economic impact. From an etiological standpoint, PRF is characterized by a polymicrobial nature involving multiple viral pathogens. While the roles of established viruses such as PCV2, PPV1, and PRRSV are well-documented, the emergence of new viruses like PCV3, PCV4, and nPPVs adds complexity to the PRF landscape. This evolving situation highlights the crucial need to establish causality in order to understand the specific contributions of these emerging viruses to PRF. Furthermore, it is essential to implement diagnostic techniques that detect the simultaneous presence of the different viruses involved in PRF cases.

Recent evidence suggests that these viral agents primarily exist in a subclinical form within the reproductive system, presenting new challenges for detection and control. These subclinical infections can result in coinfections, which are increasingly reported at the reproductive level. Future research should clarify the interactions between these viral agents and their impacts on the porcine reproductive performance.

## Figures and Tables

**Table 1:** Clinical signs associated with porcine reproductive failure caused by PCV2, PCV3, PPV1, and PRRSV infections during the three trimesters of pregnancy in gilts and sows.

	Early Pregnancy <35 Days	Mid-Pregnancy 35–70 Days	Late Pregnancy >70 Days	Reference
PCV2	Embryonic death, return to estrus	Abortions, mummified fetuses	Mummies, abortions, weak-born or non-viable piglets	[25,113,114]
PCV3	No reports	Abortions, mummified fetuses	Mummies Stillborns Abortions Weak-born piglets	[128,129,130,131]
PPV1	Embryonic death, reabsorption return to estrus	Mummies	Mummies Stillborns (rare) Healthy piglets	[107,109]
PRRSV	Embryonic death, return to estrus (rare)	Abortions, mummified fetuses	Mummies, abortions, weak-born piglets, premature farrowing	[5,132,133,134]

**Table 2:** Prevalence of emerging viral pathogens in clinical and subclinical cases of porcine reproductive failure.

Virus	Sample	Prevalence in Cases of PRF-Clinical (Reproductive Failure or SMEDI)	Prevalence in Cases of PRF-Subclinical (Lack of Reproductive Signs or SMEDI)	Reference
PCV3	Aborted fetuses	1.9–100%	2.29–59%	[50,128,129,130,139,146,148,149,150]
	Sera of sows	20–100%	13–37.5%	[26,131,139,140,151,152,153]
	PFET (paraffin- and formalin-embedded aborted tissues)	50%	Not reported	[154]
PCV4	Aborted fetuses	0.17–4.7%	Not reported	[15,44]
nPPVs	Aborted fetuses	PPV2: 2.4–23.6% PPV3: 3.2–15.8% PPV4: 7–50% PPV5: 18.8–24.2% PPV6: 18–50 PPV7: 13.2–50%	Not reported	[16,74,152,155,156,157]
	Sera of sows	PPV2 to PPV6: not reported PPV7: 24.7%	PPV2: 9.8%, PPV3: 10–40.1% PPV4: 2.5–4.2% PPV5: 20.5% PPV6: 4.5–17%, PPV7: 1.3–7.5%	[26,152,153]
	PFET (paraffin- and formalin-embedded aborted tissues)	PPV2: 96% PPV3: 38% PPV4: 6% PPV5: 28% PPV6: 57%	Not reported	[28]

**Table 3:** Histopathological lesions observed in the reproductive tissues of sows, fetuses, newborn piglets, and stillborns associated with PCV2, PCV3, PPV1, and PRRSV infections.

Virus	Source	Tissue	Lesions	Reference
PCV2	Fetuses, stillborns, and weak-born piglets	Heart Lung Liver Lymphoid tissue	Degenerated and necrotic cardiomyocytes; lymphoplasmacytic infiltration. Multifocal non-suppurative interstitial pneumonia. Hepatic congestion; non-suppurative hepatitis with periacinar necrosis. Lymphoid depletion.	[25,112,179]
PCV3	Fetuses, stillborns, and weak-born piglets	Heart Lung Kidney Brain Lymphoid tissue Placenta	Lymphocytic myocarditis (lymphoplasmacytic infiltration). Interstitial pneumonia. Interstitial nephritis (lymphoplasmacytic infiltration). Perivascular lymphocyte aggregates; gliosis. Lymphoid depletion. Necrosis and inflammation.	[50,150]
PPV1	Fetuses	Brain Heart Lung Kidney Liver Multiple organs Placenta Uterus	Perivascular accumulation of mononuclear cells (gray and white matter, meninges), inclusion bodies in cerebellum. Mononuclear infiltration in myocardium, mineralization. Mononuclear infiltration, congestion. Mononuclear cell infiltration in interstitial tissue, tubular degeneration. Mononuclear infiltration, inclusion bodies in hepatocytes, vacuolar degeneration of hepatocytes. Necrosis of cells, developing mineralization. Placentitis, calcification in allantochorion. Mononuclear infiltration, diffuse hemorrhages and edema; inclusion bodies in endometrium.	[3,176,180]
PRRSV	Stillborns and weak-born piglets	Heart Lung Liver Brain Lymphoid tissue Umbilical cord Placenta Uterus	Perivascular lymphoplasmacytic and histiocytic myocarditis; non-suppurative myocarditis. Multifocal interstitial pneumonia. Eosinophilic hepatitis, lymphoplasmacytic and histiocytic infiltration. Non-suppurative encephalitis. Lymphoid depletion; follicular atrophy. Lymphoplasmacytic and histiocytic infiltration; severe hemorrhage in the adventitia. Lymphoplasmacytic and histiocytic infiltrate in arteritis; necrosis; desquamation Lymphoplasmacytic and histiocytic myometritis	[5,172,181]

**Table 4:** Prevalence of viral coinfections associated with porcine reproductive failure: diagnostic laboratory surveillance data.

Coinfection	Sample Type	Prevalence (%)	Geographical Region
PCV2/PPV1	Abortions	1.8–2.5 2.1–10.4 4.5 20.5 7.4 5.7	Korea [25,157] Italy [16,190] Argentina [191] Colombia [26] China [192] Germany [20]
PCV2/PRRSV	Abortions	0.5 16.8 10–24 9–45	Korea [25] Colombia [26] Italy [16,190] China [192,193,194]
	Stillborns	10	Colombia [155]
PCV2/CSFV	Abortions	3.7–6.6	China [192,194]
PCV2/PRV	Abortions	1.5 9	Italy [190] China [194]
PCV3/PCV2	Abortions	13.8 26.4 5.6 4	Italy [16] Brazil [130] Colombia [26] Korea [157]
	Stillborns	5.3	Colombia [155]
	Sow sera with PRF-C	15.8 24	China [146] Colombia [155]
PCV2/nPPVs	Abortions PPv3-PPV7	2.9–5.1 16.1–50	Korea [157] Italy [16]
	Fetal tissues PPV3-PPV6 (FFEP)	5.3–62	Mexico [28]
	Sow sera	1.3–16.3	Colombia [140,153]
PCV2/PPV2	Fetal tissue (PEFF)	98	Mexico [28]
	Stillborns	5.3	Colombia [155]
PRRSV/PPV1	Abortions	0.25 1–9 1	Korea [25] Italy [16,190] Colombia [26]
PRRSV/PRV	Abortions	1 0.3–3	Italy [190] China [194]
PRRSV/EMCV	Abortions	1	Italy [190]
PRRVS/CSFV	Abortions	2.6–3.2	China [192,194]
PRRSV/nPPVs	Abortions (PPV2 to PPV7)	10–25	Italy [16]
	Sera (gilts, PPV2 to PPV7)	1.3–27	Colombia [153]
PCV3/PRRSV	Fetuses	13.8 1.9 1	Italy [16] Spain [150] Colombia [26]
PCV3/PPV1	Abortions	9 25.4 1.5	Brazil [130] Italy [16] Korea [157]
	Sow sera	50	Argentina [148]
PCV3-nPPVs	Abortions (PPV2 to PPV7)	6.7–25	Italy [16]
	Sera (gilts, PPV2 to PPV7)	0.4–15	Colombia [153]
PCV3/PPV7	Sera (sows)	34	China [152]
	Abortions	55	China [152]
PCV3/APPV	Newborn piglets (brain)	66	USA [50]
PPV1/nPPVs	Abortions PPV2 to PPV6	1.1–5.9	Korea [157]
PCV2/PRRSV/CSFV	Abortions	0.49–4	China [192,194]
PCV2/PRRSV/PCV3	Abortions	1.9 5.3	Spain [150] Colombia [155]
PCV2/PCV3/PPV1	Abortions	2 45	Colombia [26] Brazil [130]
PCV2/PCV3/PPV7	Sera (sows)	10	Colombia [140]
PCV2/PRRSV/PPV1	Abortions	4.7	Colombia [26]
PCV2/PRRSV/PPV2	Stillborns	21	Colombia [155]
PCV2/PCV4	Abortions	0.13	China [44]

**Table 5:** Observations of macroscopic and microscopic lesions, along with additional observations related to viral coinfections in porcine reproductive failure.

Coinfections	Macro Lesions	Micro Lesions	Additional Observations	Reference
PCV2/PRRSV	NR	Myocardium: mononuclear cell infiltrate, myocarditis perivascular and fibrosis	IHC: PCV2 Ag in myocardiocyte cytoplasm and lymphocytes. IHC: PRRSV Ag in lymphocytes and macrophages in myocardium.	[196]
	NR	NR	Higher PRRSV infections in PCV2-positive fetuses.	[197]
	NR	NR	Effects observed in late gestation.	[25]
	NR	No specific lesions	Lower PCV2 antibodies in PRRSV-positive pregnant sows.	[26]
PCV2/PPV1	NR	NR	Affects all pregnancy stages; weak-born piglets observed.	[25]
	Mesocolon edema, ventricular dilation,	NR	Effects observed in late gestation.	[195]
	severe visceral organ lesions (congestion, enlarged nodules, adhesions)	Intracytoplasmic inclusion bodies in macrophages, malacia foci in brain	NR	[27]
	Reduced CRL and lower fetal body weight	NR	PCV2/PPV1 coinfection: faster time to death after infection or more severe impairment of fetal development.	[20]
	NR	NR	Higher PPV1 infections in PCV2-positive gilts.	[153]
	NR	No specific lesions	Higher PCV2 viral load in PPV1-positive fetus.	[26]
PCV2/nPPVs	NR	NR	Association with aborted fetuses in PCV2/PPV5 and PCV2/PPV6 coinfections.	[28].
PCV3/PRRSV	NR	PCV3: lymphoplasmacytic infiltration in arterioles and myocarditis PRRSV: no specific lesions	Effects in late pregnancy.	[187]
PCV3/nPPVs	NR	NR	Higher PPV6 infections in PCV3-positive gilts.	[153]
PRRSV/nPPVs	NR	NR	Higher PPV5 infections in PRRSV-positive gilts.	[153]
PCV3/PPV7	NR	NR	History of severe reproductive failure. Higher PPV7 positivity in PCV3-positive sows with reproductive failure.	[152]
PCV2/PCV3/PRRSV	NR	PCV3: lymphoplasmacytic infiltration. PCV2 and PRRSV: no specific lesions.	Effects in the early and mid-pregnancy	[187] [26]
PCV2/PCV3	NR	NR	Possible inhibitory effect of PCV3 on transplacental PCV2 infection.	[20]
	NR	NR	Simultaneous detection of anti-PCV2 and anti-PCV3 antibodies in fetal tissues.	[118]

NR: not reported.

## Data Availability

All required data are available as texts and figures in the main text of this article. The sequence datasets are publicly available at NCBI GenBank.

## Supplementary Materials

The following supporting information can be downloaded at: https://www.mdpi.com/article/10.3390/v17081137/s1. Figure S1: Phylogenetic distribution of porcine circoviruses (PCVs) based on the alignment of 50 representative whole-genome nucleotide (nt) sequences from PCV1 to PCV4. Figure S2: Phylogenetic distribution of porcine parvoviruses (PPV1 through PPV8). Figure S3: Phylogenetic distribution of porcine reproductive and respiratory syndrome virus (PRRSV).

## Abbreviations

The following abbreviations are used in this manuscript:

PRF	Porcine reproductive failure
PCV2	Porcine circovirus type 2
PPV	Porcine parvovirus
PRRSV	Porcine reproductive and respiratory syndrome virus
SMEDI	Stillbirth, mummified fetuses, embryonic death, and infertility
PCV3	Porcine circovirus type 3
PCV4	Porcine circovirus type 4
nPPVs	Novel porcine parvovirus
ORF	Open reading frames
nt	Nucleotide
nspy	Nucleotide substitutions per year
RFLP	Restriction fragment length polymorphism
CRL	Crown–rump lengths
Abs	Antibodies
PCV2-RD	PCV2-reproductive disease
MDA	Maternal-derived antibodies
PCV3-RD	PCV3-reproductive disease
PCV3-SD	PCV3-systemic disease
PCV2-SI	PCV2-subclinical infection
PRF-C	clinical porcine reproductive failure
PRF-SC	subclinical porcine reproductive failure

## References

[1] Christianson W.T. (1992). Stillbirths, mummies, abortions, and early embryonic death. Vet. Clin. N. Am. Food Anim. Pract..

[2] Maes D., Peltoniemi O., Malik M. (2023). Abortion and fetal death in sows. Reprod. Domest. Anim..

[3] Streck A.F., Truyen U. (2020). Porcine Parvovirus. Curr. Issues Mol. Biol..

[4] Madson D.M., Opriessnig T. (2011). Effect of porcine circovirus type 2 (PCV2) infection on reproduction: Disease, vertical transmission, diagnostics and vaccination. Anim. Health Res. Rev..

[5] Rossow K.D. (1998). Porcine reproductive and respiratory syndrome. Vet. Pathol..

[6] Moennig V., Floegel-Niesmann G., Greiser-Wilke I. (2003). Clinical signs and epidemiology of classical swine fever: A review of new knowledge. Vet. J..

[7] Nauwynck H.J., Pensaert M.B. (1992). Abortion induced by cell-associated pseudorabies virus in vaccinated sows. Am. J. Vet. Res..

[8] Forman A.J., Pass D.A., Connaughton I.D. (1982). The characterisation and pathogenicity of porcine enteroviruses isolated in Victoria. Aust. Vet. J..

[9] Kim H.S., Christianson W.T., Joo H.S. (1991). Characterization of encephalomyocarditis virus isolated from aborted swine fetuses. Am. J. Vet. Res..

[10] Kwit K., Pomorska-Mól M., Markowska-Daniel I. (2015). Pregnancy outcome and clinical status of gilts following experimental infection by H1N2, H3N2 and H1N1pdm09 influenza A viruses during the last month of gestation. Arch. Virol..

[11] Passler T., Walz P.H. (2010). Bovine viral diarrhea virus infections in heterologous species. Anim. Health Res. Rev..

[12] Olanratmanee E.-O., Kunavongkrit A., Tummaruk P. (2010). Impact of porcine epidemic diarrhea virus infection at different periods of pregnancy on subsequent reproductive performance in gilts and sows. Anim. Reprod. Sci..

[13] Schlafer D.H., Mebus C.A. (1987). Abortion in sows experimentally infected with African swine fever virus: Pathogenesis studies. Am. J. Vet. Res..

[14] Ouyang T., Niu G., Liu X., Zhang X., Zhang Y., Ren L. (2019). Recent progress on porcine circovirus type 3. Infect. Genet. Evol..

[15] Nguyen V.-G., Do H.-Q., Huynh T.-M.-L., Park Y.-H., Park B.-K., Chung H.-C. (2022). Molecular-based detection, genetic characterization and phylogenetic analysis of porcine circovirus 4 from Korean domestic swine farms. Transbound. Emerg. Dis..

[16] Faustini G., Tucciarone C.M., Franzo G., Donneschi A., Boniotti M.B., Alborali G.L., Drigo M. (2024). Molecular Survey on Porcine Parvoviruses (PPV1-7) and Their Association with Major Pathogens in Reproductive Failure Outbreaks in Northern Italy. Viruses.

[17] Arruda B., Shen H., Zheng Y., Li G. (2021). Novel Morbillivirus as Putative Cause of Fetal Death and Encephalitis among Swine. Emerg. Infect. Dis..

[18] Schautteet K., Vanrompay D. (2011). Chlamydiaceae infections in pig. Vet. Res..

[19] Hoffmann C.W., Bilkei G. (2002). Case study: Chronic erysipelas of the sow—A subclinical manifestation of reproductive problems. Reprod. Domest. Anim..

[20] Eddicks M., Gründl J., Seifert A., Eddicks L., Reese S., Tabeling R., Swam H., Strutzberg-Minder K., Ritzmann M., Fux R. (2023). Examination on the occurrence of coinfections in diagnostic transmittals in cases of stillbirth, mummification, embryonic death, and infertility (SMEDI) syndrome in germany. Microorganisms.

[21] Rebollada-Merino A., García-Seco T., Pérez-Sancho M., Domínguez L., Rodríguez-Bertos A. (2023). Histopathologic and immunohistochemical findings in the placentas and fetuses of domestic swine naturally infected with Brucella suis biovar 2. J. Vet. Diagn. Investig..

[22] Zhang N., Huang D., Wu W., Liu J., Liang F., Zhou B., Guan P. (2018). Animal brucellosis control or eradication programs worldwide: A systematic review of experiences and lessons learned. Prev. Vet. Med..

[23] Moreno L.Z., Matajira C.E.C., Poor A.P., Mesquita R.E., Gomes V.T.M., Silva A.P.S., Amigo C.R., Christ A.P.G., Barbosa M.R.F., Sato M.I.Z. (2018). Identification through MALDI-TOF mass spectrometry and antimicrobial susceptibility profiling of bacterial pathogens isolated from sow urinary tract infection. Vet. Q..

[24] Donneschi A., Recchia M., Romeo C., Pozzi P., Salogni C., Maisano A.M., Santucci G., Scali F., Faccini S., Boniotti M.B. (2024). Infectious Agents Associated with Abortion Outbreaks in Italian Pig Farms from 2011 to 2021. Vet. Sci..

[25] Kim J., Jung K., Chae C. (2004). Prevalence of porcine circovirus type 2 in aborted fetuses and stillborn piglets. Vet. Rec..

[26] Vargas-Bermudez D.S., Polo G., Mogollon J.D., Jaime J. (2025). Longitudinal Monitoring of Mono- and Coinfections Involving Primary Porcine Reproductive Viruses (PCV2, PPV1, and PRRSV) as Well as Emerging Viruses (PCV3, PCV4, and nPPVs) in Primiparous and Multiparous Sows and Their Litters. Pathogens.

[27] Sharma R., Saikumar G. (2010). Porcine parvovirus- and porcine circovirus 2-associated reproductive failure and neonatal mortality in crossbred Indian pigs. Trop. Anim. Health Prod..

[28] Garcia-Camacho L.A., Vargas-Ruiz A., Marin-Flamand E., Ramírez-Álvarez H., Brown C. (2020). A retrospective study of DNA prevalence of porcine parvoviruses in Mexico and its relationship with porcine circovirus associated disease. Microbiol. Immunol..

[29] Varsani A., Harrach B., Roumagnac P., Benkő M., Breitbart M., Delwart E., Franzo G., Kazlauskas D., Rosario K., Segalés J. (2024). 2024 taxonomy update for the family Circoviridae. Arch. Virol..

[30] Tischer I., Gelderblom H., Vettermann W., Koch M.A. (1982). A very small porcine virus with circular single-stranded DNA. Nature.

[31] Fenaux M., Halbur P.G., Gill M., Toth T.E., Meng X.J. (2000). Genetic characterization of type 2 porcine circovirus (PCV-2) from pigs with postweaning multisystemic wasting syndrome in different geographic regions of North America and development of a differential PCR-restriction fragment length polymorphism assay to detect and differentiate between infections with PCV-1 and PCV-2. J. Clin. Microbiol..

[32] Palinski R., Piñeyro P., Shang P., Yuan F., Guo R., Fang Y., Byers E., Hause B.M. (2017). A Novel Porcine Circovirus Distantly Related to Known Circoviruses Is Associated with Porcine Dermatitis and Nephropathy Syndrome and Reproductive Failure. J. Virol..

[33] Tian R.-B., Zhao Y., Cui J.-T., Zheng H.-H., Xu T., Hou C.-Y., Wang Z.-Y., Li X.-S., Zheng L.-L., Chen H.-Y. (2021). Molecular detection and phylogenetic analysis of Porcine circovirus 4 in Henan and Shanxi Provinces of China. Transbound. Emerg. Dis..

[34] Breitbart M., Delwart E., Rosario K., Segalés J., Varsani A. (2017). Ictv Report Consortium ICTV virus taxonomy profile: Circoviridae. J. Gen. Virol..

[35] Hamel A.L., Lin L.L., Nayar G.P. (1998). Nucleotide sequence of porcine circovirus associated with postweaning multisystemic wasting syndrome in pigs. J. Virol..

[36] Tischer I., Mields W., Wolff D., Vagt M., Griem W. (1986). Studies on epidemiology and pathogenicity of porcine circovirus. Arch. Virol..

[37] Ellis J., Hassard L., Clark E., Harding J., Allan G., Willson P., Strokappe J., Martin K., McNeilly F., Meehan B. (1998). Isolation of circovirus from lesions of pigs with postweaning multisystemic wasting syndrome. Can. Vet. J..

[38] Segalés J. (2012). Porcine circovirus type 2 (PCV2) infections: Clinical signs, pathology and laboratory diagnosis. Virus Res..

[39] Wang Y., Noll L., Lu N., Porter E., Stoy C., Zheng W., Liu X., Peddireddi L., Niederwerder M., Bai J. (2020). Genetic diversity and prevalence of porcine circovirus type 3 (PCV3) and type 2 (PCV2) in the Midwest of the USA during 2016-2018. Transbound. Emerg. Dis..

[40] Franzo G., Segalés J., Sola C. (2018). Porcine circovirus 2 (PCV-2) genotype update and proposal of a new genotyping methodology. PLoS ONE.

[41] Franzo G., Delwart E., Fux R., Hause B., Su S., Zhou J., Segalés J. (2020). Genotyping Porcine Circovirus 3 (PCV-3) Nowadays: Does It Make Sense?. Viruses.

[42] Zhang H.-H., Hu W.-Q., Li J.-Y., Liu T.-N., Zhou J.-Y., Opriessnig T., Xiao C.-T. (2020). Novel circovirus species identified in farmed pigs designated as Porcine circovirus 4, Hunan province, China. Transbound. Emerg. Dis..

[43] Kim D.-Y., Kim H.-R., Park J.-H., Kwon N.-Y., Kim J.-M., Kim J.-K., Park J.-H., Lee K.-K., Kim S.-H., Kim W.-I. (2022). Detection of a novel porcine circovirus 4 in Korean pig herds using a loop-mediated isothermal amplification assay. J. Virol. Methods.

[44] Sirisereewan C., Nguyen T.C., Piewbang C., Jittimanee S., Kedkovid R., Thanawongnuwech R. (2023). Molecular detection and genetic characterization of porcine circovirus 4 (PCV4) in Thailand during 2019–2020. Sci. Rep..

[45] Holgado-Martín R., Arnal J.L., Sibila M., Franzo G., Martín-Jurado D., Risco D., Segalés J., Gómez L. (2023). First detection of porcine circovirus 4 (PCV-4) in Europe. Virol. J..

[46] Kroeger M., Vargas-Bermudez D.S., Jaime J., Parada J., Groeltz J., Gauger P., Piñeyro P. (2024). First detection of PCV4 in swine in the United States: Codetection with PCV2 and PCV3 and direct detection within tissues. Sci. Rep..

[47] Xu T., Hou C.-Y., Zhang Y.-H., Li H.-X., Chen X.-M., Pan J.-J., Chen H.-Y. (2022). Simultaneous detection and genetic characterization of porcine circovirus 2 and 4 in Henan province of China. Gene.

[48] Klaumann F., Correa-Fiz F., Franzo G., Sibila M., Núñez J.I., Segalés J. (2018). Current Knowledge on Porcine circovirus 3 (PCV-3): A Novel Virus With a Yet Unknown Impact on the Swine Industry. Front. Vet. Sci..

[49] Tamura K., Stecher G., Kumar S. (2021). MEGA11: Molecular Evolutionary Genetics Analysis version 11. Mol. Biol. Evol..

[50] Arruda B., Piñeyro P., Derscheid R., Hause B., Byers E., Dion K., Long D., Sievers C., Tangen J., Williams T. (2019). PCV3-associated disease in the United States swine herd. Emerg. Microbes Infect..

[51] Vargas-Bermudez D.S., Mogollon J.D., Franco-Rodriguez C., Jaime J. (2023). The novel porcine parvoviruses: Current state of knowledge and their possible implications in clinical syndromes in pigs. Viruses.

[52] Pénzes J.J., de Souza W.M., Agbandje-McKenna M., Gifford R.J. (2019). An Ancient Lineage of Highly Divergent Parvoviruses Infects both Vertebrate and Invertebrate Hosts. Viruses.

[53] Cotmore S.F., Agbandje-McKenna M., Canuti M., Chiorini J.A., Eis-Hubinger A.-M., Hughes J., Mietzsch M., Modha S., Ogliastro M., Pénzes J.J. (2019). Ictv Report Consortium ICTV virus taxonomy profile: Parvoviridae. J. Gen. Virol..

[54] Sol N., Le Junter J., Vassias I., Freyssinier J.M., Thomas A., Prigent A.F., Rudkin B.B., Fichelson S., Morinet F. (1999). Possible interactions between the NS-1 protein and tumor necrosis factor alpha pathways in erythroid cell apoptosis induced by human parvovirus B19. J. Virol..

[55] Mietzsch M., Pénzes J.J., Agbandje-McKenna M. (2019). Twenty-Five Years of Structural Parvovirology. Viruses.

[56] Cadar D., Dán Á., Tombácz K., Lőrincz M., Kiss T., Becskei Z., Spînu M., Tuboly T., Cságola A. (2012). Phylogeny and evolutionary genetics of porcine parvovirus in wild boars. Infect. Genet. Evol..

[57] Vargas-Bermudez D.S., Prandi B.A., de Souza U.J.B., Durães-Carvalho R., Mogollón J.D., Campos F.S., Roehe P.M., Jaime J. (2024). Molecular Epidemiology and Phyloevolutionary Analysis of Porcine Parvoviruses (PPV1 through PPV7) Detected in Replacement Gilts from Colombia. Int. J. Mol. Sci..

[58] Hijikata M., Abe K., Win K.M., Shimizu Y.K., Keicho N., Yoshikura H. (2001). Identification of new parvovirus DNA sequence in swine sera from Myanmar. Jpn. J. Infect. Dis..

[59] Saekhow P., Mawatari T., Ikeda H. (2014). Coexistence of multiple strains of porcine parvovirus 2 in pig farms. Microbiol. Immunol..

[60] Cadar D., Lőrincz M., Kiss T., Novosel D., Podgorska K., Becskei Z., Tuboly T., Cságola A. (2013). Emerging novel porcine parvoviruses in Europe: Origin, evolution, phylodynamics and phylogeography. J. Gen. Virol..

[61] Afolabi K.O., Iweriebor B.C., Obi L.C., Okoh A.I. (2019). Prevalence of porcine parvoviruses in some South African swine herds with background of porcine circovirus type 2 infection. Acta Trop..

[62] Lau S.K.P., Woo P.C.Y., Tse H., Fu C.T.Y., Au W.-K., Chen X.-C., Tsoi H.-W., Tsang T.H.F., Chan J.S.Y., Tsang D.N.C. (2008). Identification of novel porcine and bovine parvoviruses closely related to human parvovirus 4. J. Gen. Virol..

[63] Cságola A., Lőrincz M., Cadar D., Tombácz K., Biksi I., Tuboly T. (2012). Detection, prevalence and analysis of emerging porcine parvovirus infections. Arch. Virol..

[64] Li S., Wei Y., Liu J., Tang Q., Liu C. (2013). Prevalence of porcine hokovirus and its co-infection with porcine circovirus 2 in China. Arch. Virol..

[65] Xiao C.-T., Giménez-Lirola L.G., Halbur P.G., Opriessnig T. (2012). Increasing porcine PARV4 prevalence with pig age in the U.S. pig population. Vet. Microbiol..

[66] Adlhoch C., Kaiser M., Kingsley M.T., Schwarz N.G., Ulrich M., de Paula V.S., Ehlers J., Löwa A., Daniel A.M., Poppert S. (2013). Porcine hokovirus in domestic pigs, Cameroon. Emerg. Infect. Dis..

[67] Cheung A.K., Wu G., Wang D., Bayles D.O., Lager K.M., Vincent A.L. (2010). Identification and molecular cloning of a novel porcine parvovirus. Arch. Virol..

[68] Kim S.-C., Kim J.-H., Kim J.-Y., Park G.-S., Jeong C.-G., Kim W.-I. (2022). Prevalence of porcine parvovirus 1 through 7 (PPV1-PPV7) and co-factor association with PCV2 and PRRSV in Korea. BMC Vet. Res..

[69] Xiao C.-T., Halbur P.G., Opriessnig T. (2013). Complete genome sequence of a novel porcine parvovirus (PPV) provisionally designated PPV5. Genome Announc..

[70] Xiao C.-T., Giménez-Lirola L.G., Jiang Y.-H., Halbur P.G., Opriessnig T., Subbiah E. (2013). Characterization of a novel porcine parvovirus tentatively designated PPV5. PLoS ONE.

[71] Wu R., Wen Y., Huang X., Wen X., Yan Q., Huang Y., Ma X., Cao S. (2014). First complete genomic characterization of a porcine parvovirus 5 isolate from China. Arch. Virol..

[72] Miłek D., Woźniak A., Guzowska M., Stadejek T. (2019). Detection Patterns of Porcine Parvovirus (PPV) and Novel Porcine Parvoviruses 2 through 6 (PPV2-PPV6) in Polish Swine Farms. Viruses.

[73] Cibulski S., Alves de Lima D., Fernandes Dos Santos H., Teixeira T.F., Tochetto C., Mayer F.Q., Roehe P.M. (2021). A plate of viruses: Viral metagenomics of supermarket chicken, pork and beef from Brazil. Virology.

[74] Ni J., Qiao C., Han X., Han T., Kang W., Zi Z., Cao Z., Zhai X., Cai X. (2014). Identification and genomic characterization of a novel porcine parvovirus (PPV6) in China. Virol. J..

[75] Schirtzinger E.E., Suddith A.W., Hause B.M., Hesse R.A. (2015). First identification of porcine parvovirus 6 in North America by viral metagenomic sequencing of serum from pigs infected with porcine reproductive and respiratory syndrome virus. Virol. J..

[76] Cui J., Fan J., Gerber P.F., Biernacka K., Stadejek T., Xiao C.-T., Opriessnig T. (2017). First identification of porcine parvovirus 6 in Poland. Virus Genes..

[77] Palinski R.M., Mitra N., Hause B.M. (2016). Discovery of a novel Parvovirinae virus, porcine parvovirus 7, by metagenomic sequencing of porcine rectal swabs. Virus Genes..

[78] Xing X., Zhou H., Tong L., Chen Y., Sun Y., Wang H., Zhang G. (2018). First identification of porcine parvovirus 7 in China. Arch. Virol..

[79] Miłek D., Woźniak A., Stadejek T. (2018). The detection and genetic diversity of novel porcine parvovirus 7 (PPV7) on Polish pig farms. Res. Vet. Sci..

[80] Park G.-N., Song S., Cha R.M., Choe S., Shin J., Kim S.-Y., Hyun B.-H., Park B.-K., An D.-J. (2021). Genetic analysis of porcine parvoviruses detected in South Korean wild boars. Arch. Virol..

[81] Li J., Xiao Y., Qiu M., Li X., Li S., Lin H., Li X., Zhu J., Chen N., Jones C.J. (2021). A Systematic Investigation Unveils High Coinfection Status of Porcine Parvovirus Types 1 through 7 in China from 2016 to 2020. Microbiol. Spectr..

[82] Guo Y., Yan G., Chen S., Han H., Li J., Zhang H., Luo S., Liu M., Wu Q., Li Q. (2022). Identification and genomic characterization of a novel porcine parvovirus in China. Front. Vet. Sci..

[83] Igriczi B., Dénes L., Schönhardt K., Balka G. (2024). First report of porcine parvovirus 8 in europe: Widespread detection and genetic characterization on commercial pig farms in hungary and slovakia. Animals.

[84] Vargas-Bermudez D.S., Jaime J. (2024). The first report of porcine parvovirus 8 (PPV8) on the American continent is associated with pigs in Colombia with porcine respiratory disease. Arch. Virol..

[85] Brinton M.A., Gulyaeva A.A., Balasuriya U.B.R., Dunowska M., Faaberg K.S., Goldberg T., Leung F.C.C., Nauwynck H.J., Snijder E.J., Stadejek T. (2021). ICTV virus taxonomy profile: Arteriviridae 2021. J. Gen. Virol..

[86] Benfield D.A., Nelson E., Collins J.E., Harris L., Goyal S.M., Robison D., Christianson W.T., Morrison R.B., Gorcyca D., Chladek D. (1992). Characterization of swine infertility and respiratory syndrome (SIRS) virus (isolate ATCC VR-2332). J. Vet. Diagn. Investig..

[87] Conzelmann K.K., Visser N., Van Woensel P., Thiel H.J. (1993). Molecular characterization of porcine reproductive and respiratory syndrome virus, a member of the arterivirus group. Virology.

[88] Music N., Gagnon C.A. (2010). The role of porcine reproductive and respiratory syndrome (PRRS) virus structural and non-structural proteins in virus pathogenesis. Anim. Health Res. Rev..

[89] Shi M., Lam T.T.-Y., Hon C.-C., Murtaugh M.P., Davies P.R., Hui R.K.-H., Li J., Wong L.T.-W., Yip C.-W., Jiang J.-W. (2010). Phylogeny-based evolutionary, demographical, and geographical dissection of North American type 2 porcine reproductive and respiratory syndrome viruses. J. Virol..

[90] Kim W.-I., Kim J.-J., Cha S.-H., Wu W.-H., Cooper V., Evans R., Choi E.-J., Yoon K.-J. (2013). Significance of genetic variation of PRRSV ORF5 in virus neutralization and molecular determinants corresponding to cross neutralization among PRRS viruses. Vet. Microbiol..

[91] Stadejek T., Stankevicius A., Murtaugh M.P., Oleksiewicz M.B. (2013). Molecular evolution of PRRSV in Europe: Current state of play. Vet. Microbiol..

[92] Kapur V., Elam M.R., Pawlovich T.M., Murtaugh M.P. (1996). Genetic variation in porcine reproductive and respiratory syndrome virus isolates in the midwestern United States. J. Gen. Virol..

[93] Wesley R.D., Mengeling W.L., Lager K.M., Clouser D.F., Landgraf J.G., Frey M.L. (1998). Differentiation of a porcine reproductive and respiratory syndrome virus vaccine strain from North American field strains by restriction fragment length polymorphism analysis of ORF 5. J. Vet. Diagn. Investig..

[94] Paploski I.A.D., Corzo C., Rovira A., Murtaugh M.P., Sanhueza J.M., Vilalta C., Schroeder D.C., VanderWaal K. (2019). Temporal Dynamics of Co-circulating Lineages of Porcine Reproductive and Respiratory Syndrome Virus. Front. Microbiol..

[95] Kikuti M., Paploski I.A.D., Pamornchainavakul N., Picasso-Risso C., Schwartz M., Yeske P., Leuwerke B., Bruner L., Murray D., Roggow B.D. (2021). Emergence of a new lineage 1C variant of porcine reproductive and respiratory syndrome virus 2 in the united states. Front. Vet. Sci..

[96] Cha S.-H., Chang C.-C., Yoon K.-J. (2004). Instability of the restriction fragment length polymorphism pattern of open reading frame 5 of porcine reproductive and respiratory syndrome virus during sequential pig-to-pig passages. J. Clin. Microbiol..

[97] Murtaugh M.P., Stadejek T., Abrahante J.E., Lam T.T.Y., Leung F.C.-C. (2010). The ever-expanding diversity of porcine reproductive and respiratory syndrome virus. Virus Res..

[98] Wang A., Chen Q., Wang L., Madson D., Harmon K., Gauger P., Zhang J., Li G. (2019). Recombination between Vaccine and Field Strains of Porcine Reproductive and Respiratory Syndrome Virus. Emerg. Infect. Dis..

[99] Wang X., Marthaler D., Rovira A., Rossow S., Murtaugh M.P. (2015). Emergence of a virulent porcine reproductive and respiratory syndrome virus in vaccinated herds in the United States. Virus Res..

[100] Jeong J., Kang I., Park C., Kim S., Park S.-J., Park K.H., Oh T., Yang S., Yoon J.S., Lee O. (2018). A comparison of the severity of reproductive failure between single and dual infection with porcine reproductive and respiratory syndrome virus (PRRSV)-1 and PRRSV-2 in late-term pregnancy gilts. Transbound. Emerg. Dis..

[101] Mengeling W.L., Vorwald A.C., Lager K.M., Brockmeier S.L. (1996). Comparison among strains of porcine reproductive and respiratory syndrome virus for their ability to cause reproductive failure. Am. J. Vet. Res..

[102] Halbur P.G., Paul P.S., Frey M.L., Landgraf J., Eernisse K., Meng X.J., Lum M.A., Andrews J.J., Rathje J.A. (1995). Comparison of the pathogenicity of two US porcine reproductive and respiratory syndrome virus isolates with that of the Lelystad virus. Vet. Pathol..

[103] Opriessnig T., Halbur P.G., Yoon K.J., Pogranichniy R.M., Harmon K.M., Evans R., Key K.F., Pallares F.J., Thomas P., Meng X.J. (2002). Comparison of molecular and biological characteristics of a modified live porcine reproductive and respiratory syndrome virus (PRRSV) vaccine (ingelvac PRRS MLV), the parent strain of the vaccine (ATCC VR2332), ATCC VR2385, and two recent field isolates of PRRSV. J. Virol..

[104] Mengeling W.L., Lager K.M., Vorwald A.C. (1998). Clinical consequences of exposing pregnant gilts to strains of porcine reproductive and respiratory syndrome (PRRS) virus isolated from field cases of “atypical” PRRS. Am. J. Vet. Res..

[105] Linhares D.C.L., Betlach C., Morrison R.B. (2017). Effect of immunologic solutions on sows and gilts on time to stability, and production losses in breeding herds infected with 1-7-4 PRRSv. Prev. Vet. Med..

[106] Karniychuk U.U., Saha D., Geldhof M., Vanhee M., Cornillie P., Van den Broeck W., Nauwynck H.J. (2011). Porcine reproductive and respiratory syndrome virus (PRRSV) causes apoptosis during its replication in fetal implantation sites. Microb. Pathog..

[107] Mengeling W.L., Lager K.M., Vorwald A.C. (2000). The effect of porcine parvovirus and porcine reproductive and respiratory syndrome virus on porcine reproductive performance. Anim. Reprod. Sci..

[108] Zhao H., Zhao G., Wang W. (2016). Susceptibility of porcine preimplantation embryos to viruses associated with reproductive failure. Theriogenology.

[109] Choi C.S., Molitor T.W., Joo H.S., Gunther R. (1987). Pathogenicity of a skin isolate of porcine parvovirus in swine fetuses. Vet. Microbiol..

[110] Sanchez R.E., Nauwynck H.J., McNeilly F., Allan G.M., Pensaert M.B. (2001). Porcine circovirus 2 infection in swine foetuses inoculated at different stages of gestation. Vet. Microbiol..

[111] Karniychuk U.U., Nauwynck H.J. (2013). Pathogenesis and prevention of placental and transplacental porcine reproductive and respiratory syndrome virus infection. Vet. Res..

[112] O’Connor B., Gauvreau H., West K., Bogdan J., Ayroud M., Clark E.G., Konoby C., Allan G., Ellis J.A. (2001). Multiple porcine circovirus 2-associated abortions and reproductive failure in a multisite swine production unit. Can. Vet. J..

[113] Mateusen B., Maes D.G.D., Van Soom A., Lefebvre D., Nauwynck H.J. (2007). Effect of a porcine circovirus type 2 infection on embryos during early pregnancy. Theriogenology.

[114] Johnson C.S., Joo H.S., Direksin K., Yoon K.-J., Choi Y.K. (2002). Experimental in utero inoculation of late-term swine fetuses with porcine circovirus type 2. J. Vet. Diagn. Investig..

[115] Pensaert M.B., Sanchez R.E., Ladekjaer-Mikkelsen A.S., Allan G.M., Nauwynck H.J. (2004). Viremia and effect of fetal infection with porcine viruses with special reference to porcine circovirus 2 infection. Vet. Microbiol..

[116] Gerber P.F., Garrocho F.M., Lana A.M.Q., Lobato Z.I.P. (2012). Fetal infections and antibody profiles in pigs naturally infected with porcine circovirus type 2 (PCV2). Can. J. Vet. Res..

[117] Madson D.M., Patterson A.R., Ramamoorthy S., Pal N., Meng X.J., Opriessnig T. (2009). Reproductive failure experimentally induced in sows via artificial insemination with semen spiked with porcine circovirus type 2. Vet. Pathol..

[118] Hernández J., Henao-Díaz A., Reséndiz-Sandoval M., Ramírez-Morán J., Cota-Valdez A., Mata-Haro V., Giménez-Lirola L.G. (2025). Evaluation of IgM, IgA, and IgG Antibody Responses Against PCV3 and PCV2 in Tissues of Aborted Fetuses from Late-Term Co-Infected Sows. Pathogens.

[119] Calsamiglia M., Fraile L., Espinal A., Cuxart A., Seminati C., Martín M., Mateu E., Domingo M., Segalés J. (2007). Sow porcine circovirus type 2 (PCV2) status effect on litter mortality in postweaning multisystemic wasting syndrome (PMWS). Res. Vet. Sci..

[120] Ladekjaer-Mikkelsen A.S., Nielsen J., Storgaard T., Bøtner A., Allan G., McNeilly F. (2001). Transplacental infection with PCV-2 associated with reproductive failure in a gilt. Vet. Rec..

[121] Pittman J.S. (2008). Reproductive failure associated with porcine circovirus type 2 in gilts. J. Swine Health Prod..

[122] Eddicks M., Koeppen M., Willi S., Fux R., Reese S., Sutter G., Stadler J., Ritzmann M. (2016). Low prevalence of porcine circovirus type 2 infections in farrowing sows and corresponding pre-suckling piglets in southern German pig farms. Vet. Microbiol..

[123] Opriessnig T., Meng X.-J., Halbur P.G. (2007). Porcine circovirus type 2 associated disease: Update on current terminology, clinical manifestations, pathogenesis, diagnosis, and intervention strategies. J. Vet. Diagn. Investig..

[124] Dvorak C.M.T., Payne B.J., Seate J.L., Murtaugh M.P. (2018). Effect of maternal antibody transfer on antibody dynamics and control of porcine circovirus type 2 infection in offspring. Viral Immunol..

[125] Gerber P.F., Garrocho F.M., Lana A.M.Q., Lobato Z.I.P. (2011). Serum antibodies and shedding of infectious porcine circovirus 2 into colostrum and milk of vaccinated and unvaccinated naturally infected sows. Vet. J..

[126] Hansen M.S., Hjulsager C.K., Bille-Hansen V., Haugegaard S., Dupont K., Høgedal P., Kunstmann L., Larsen L.E. (2010). Selection of method is crucial for the diagnosis of porcine circovirus type 2 associated reproductive failures. Vet. Microbiol..

[127] Segalés J., Sibila M. (2022). Revisiting porcine circovirus disease diagnostic criteria in the current porcine circovirus 2 epidemiological context. Vet. Sci..

[128] Faccini S., Barbieri I., Gilioli A., Sala G., Gibelli L.R., Moreno A., Sacchi C., Rosignoli C., Franzini G., Nigrelli A. (2017). Detection and genetic characterization of Porcine circovirus type 3 in Italy. Transbound. Emerg. Dis..

[129] Ruiz A., Saporiti V., Huerta E., Balasch M., Segalés J., Sibila M. (2022). Exploratory Study of the Frequency of Detection and Tissue Distribution of Porcine Circovirus 3 (PCV-3) in Pig Fetuses at Different Gestational Ages. Pathogens.

[130] Dal Santo A.C., Cezario K.C., Bennemann P.E., Machado S.A., Martins M. (2020). Full-genome sequences of porcine circovirus 3 (PCV3) and high prevalence in mummified fetuses from commercial farms in Brazil. Microb. Pathog..

[131] Tochetto C., Alves de Lima D., Muterle Varela A.P., Ortiz L.C., Loiko M.R., Scheffer C.M., Paim W.P., Cibulski S.P., Cerva C., Herpich J. (2020). Investigation on porcine circovirus type 3 in serum of farrowing sows with stillbirths. Microb. Pathog..

[132] Unterweger C., Kreutzmann H., Buenger M., Klingler E., Auer A., Rümenapf T., Truyen U., Ladinig A. (2023). Litters of Various-Sized Mummies (LVSM) and Stillborns after Porcine Reproductive and Respiratory Syndrome Virus Type 1 Infection-A Case Report. Vet. Sci..

[133] Christianson W.T., Choi C.S., Collins J.E., Molitor T.W., Morrison R.B., Joo H.S. (1993). Pathogenesis of porcine reproductive and respiratory syndrome virus infection in mid-gestation sows and fetuses. Can. J. Vet. Res..

[134] Prieto C., Suárez P., Simarro I., García C., Fernández A., Castro J.M. (1997). Transplacental infection following exposure of gilts to porcine reproductive and respiratory syndrome virus at the onset of gestation. Vet. Microbiol..

[135] Kim S.-C., Nazki S., Kwon S., Juhng J.-H., Mun K.-H., Jeon D.-Y., Jeong C.-G., Khatun A., Kang S.-J., Kim W.-I. (2018). The prevalence and genetic characteristics of porcine circovirus type 2 and 3 in Korea. BMC Vet. Res..

[136] Igriczi B., Dénes L., Biksi I., Albert E., Révész T., Balka G. (2022). High prevalence of porcine circovirus 3 in hungarian pig herds: Results of a systematic sampling protocol. Viruses.

[137] Tochetto C., Lima D.A., Varela A.P.M., Loiko M.R., Paim W.P., Scheffer C.M., Herpich J.I., Cerva C., Schmitd C., Cibulski S.P. (2018). Full-Genome Sequence of Porcine Circovirus type 3 recovered from serum of sows with stillbirths in Brazil. Transbound. Emerg. Dis..

[138] Guo Z., Li X., Deng R., Zhang G. (2019). Detection and genetic characteristics of porcine circovirus 3 based on oral fluids from asymptomatic pigs in central China. BMC Vet. Res..

[139] Zou Y., Zhang N., Zhang J., Zhang S., Jiang Y., Wang D., Tan Q., Yang Y., Wang N. (2018). Molecular detection and sequence analysis of porcine circovirus type 3 in sow sera from farms with prolonged histories of reproductive problems in Hunan, China. Arch. Virol..

[140] Vargas-Bermúdez D.S., Vargas-Pinto M.A., Mogollón J.D., Jaime J. (2021). Field infection of a gilt and its litter demonstrates vertical transmission and effect on reproductive failure caused by porcine circovirus type 3 (PCV3). BMC Vet. Res..

[141] Deim Z., Dencső L., Erdélyi I., Valappil S.K., Varga C., Pósa A., Makrai L., Rákhely G. (2019). Porcine circovirus type 3 detection in a Hungarian pig farm experiencing reproductive failures. Vet. Rec..

[142] Reséndiz-Sandoval M., Vázquez-García V.A., Contreras-Vega K., Melgoza-González E.A., Mata-Haro V., Gimenez-Lirola L., Hernández J. (2023). A Retrospective Analysis of Porcine Circovirus Type 3 in Samples Collected from 2008 to 2021 in Mexico. Viruses.

[143] Saporiti V., Franzo G., Sibila M., Segalés J. (2021). Porcine circovirus 3 (PCV-3) as a causal agent of disease in swine and a proposal of PCV-3 associated disease case definition. Transbound. Emerg. Dis..

[144] Cobos À., Ruiz A., Pérez M., Llorens A., Huerta E., Correa-Fiz F., Lohse R., Balasch M., Segalés J., Sibila M. (2023). Experimental Inoculation of Porcine Circovirus 3 (PCV-3) in Pregnant Gilts Causes PCV-3-Associated Lesions in Newborn Piglets that Persist until Weaning. Transbound. Emerg. Dis..

[145] Kedkovid R., Woonwong Y., Arunorat J., Sirisereewan C., Sangpratum N., Kesdangsakonwut S., Tummaruk P., Teankum K., Assavacheep P., Jittimanee S. (2018). Porcine circovirus type 3 (PCV3) shedding in sow colostrum. Vet. Microbiol..

[146] Ku X., Chen F., Li P., Wang Y., Yu X., Fan S., Qian P., Wu M., He Q. (2017). Identification and genetic characterization of porcine circovirus type 3 in China. Transbound. Emerg. Dis..

[147] Kroeger M., Temeeyasen G., Dilberger-Lawson S., Nelson E., Magtoto R., Gimenez-Lirola L., Piñeyro P., Rajao D.S. (2024). The porcine circovirus 3 humoral response: Characterization of maternally derived antibodies and dynamic following experimental infection. Microbiol. Spectr..

[148] Serena M.S., Cappuccio J.A., Barrales H., Metz G.E., Aspitia C.G., Lozada I., Perfumo C.J., Quiroga M.A., Piñeyro P., Echeverría M.G. (2021). First detection and genetic characterization of porcine circovirus type 3 (PCV3) in Argentina and its association with reproductive failure. Transbound. Emerg. Dis..

[149] Zheng S., Wu X., Zhang L., Xin C., Liu Y., Shi J., Peng Z., Xu S., Fu F., Yu J. (2017). The occurrence of porcine circovirus 3 without clinical infection signs in Shandong Province. Transbound. Emerg. Dis..

[150] Saporiti V., Valls L., Maldonado J., Perez M., Correa-Fiz F., Segalés J., Sibila M. (2021). Porcine Circovirus 3 Detection in Aborted Fetuses and Stillborn Piglets from Swine Reproductive Failure Cases. Viruses.

[151] Yuzhakov A.G., Raev S.A., Alekseev K.P., Grebennikova T.V., Verkhovsky O.A., Zaberezhny A.D., Aliper T.I. (2018). First detection and full genome sequence of porcine circovirus type 3 in Russia. Virus Genes..

[152] Mai J., Wang D., Zou Y., Zhang S., Meng C., Wang A., Wang N. (2021). High Co-infection Status of Novel Porcine Parvovirus 7 With Porcine Circovirus 3 in Sows That Experienced Reproductive Failure. Front. Vet. Sci..

[153] Vargas-Bermudez D.S., Diaz A., Polo G., Mogollon J.D., Jaime J. (2024). Infection and Coinfection of Porcine-Selected Viruses (PPV1 to PPV8, PCV2 to PCV4, and PRRSV) in Gilts and Their Associations with Reproductive Performance. Vet. Sci..

[154] Cobos À., Sibila M., Alomar J., Pérez M., Huerta E., Segalés J. (2022). Retrospective assessment of porcine circovirus 3 (PCV-3) in formalin-fixed, paraffin-embedded tissues from pigs affected by different clinical-pathological conditions. Porc. Health Manag..

[155] Vargas-Bermudez D.S., Mainenti M., Naranjo-Ortiz M.F., Mogollon J.D., Piñeyro P., Jaime J., Chen N. (2024). First Report of Porcine Parvovirus 2 (PPV2) in Pigs from Colombia Associated with Porcine Reproductive Failure (PRF) and Porcine Respiratory Disease Complex (PRDC). Transbound. Emerg. Dis..

[156] Lagan Tregaskis P., Staines A., Gordon A., Sheridan P., McMenamy M., Duffy C., Collins P.J., Mooney M.H., Lemon K. (2021). Co-infection status of novel parvovirus’s (PPV2 to 4) with porcine circovirus 2 in porcine respiratory disease complex and porcine circovirus-associated disease from 1997 to 2012. Transbound. Emerg. Dis..

[157] Ouh I.-O., Lee J.-Y., Choi H., Moon S.Y., Song J.Y., Hyun B.-H., Kwak D., Lee Y.-H., Park C.-K. (2023). Prevalence of Porcine Parvoviruses 1 to 6 and Porcine Circovirus 3 Infections and of Their Co-infections With Porcine Circovirus 2 in the Republic of Korea. Preprints.

[158] Hou C.-Y., Zhang L.-H., Zhang Y.-H., Cui J.-T., Zhao L., Zheng L.-L., Chen H.-Y. (2022). Phylogenetic analysis of porcine circovirus 4 in Henan Province of China: A retrospective study from 2011 to 2021. Transbound. Emerg. Dis..

[159] Ge M., Hu W.-Q., Ning K.-M., Li S.-Y., Xiao C.-T. (2021). The seroprevalence of the newly identified porcine circovirus type 4 in China investigated by an enzymed-linked immunosorbent assay. Transbound. Emerg. Dis..

[160] Opriessnig T., Fenaux M., Yu S., Evans R.B., Cavanaugh D., Gallup J.M., Pallares F.J., Thacker E.L., Lager K.M., Meng X.J. (2004). Effect of porcine parvovirus vaccination on the development of PMWS in segregated early weaned pigs coinfected with type 2 porcine circovirus and porcine parvovirus. Vet. Microbiol..

[161] Mészáros I., Olasz F., Cságola A., Tijssen P., Zádori Z. (2017). Biology of Porcine Parvovirus (*Ungulate parvovirus* 1). Viruses.

[162] Gava D., Souza C.K., Mores T.J., Argenti L.E., Streck A.F., Canal C.W., Bortolozzo F.P., Wentz I. (2017). Dynamics of vanishing of maternally derived antibodies of Ungulate protoparvovirus 1 suggests an optimal age for gilts vaccination. Trop. Anim. Health Prod..

[163] Joo H.S., Donaldson-Wood C.R., Johnson R.H. (1976). Observations on the pathogenesis of porcine parvovirus infection. Arch. Virol..

[164] Zeeuw E.J.L., Leinecker N., Herwig V., Selbitz H.J., Truyen U. (2007). Study of the virulence and cross-neutralization capability of recent porcine parvovirus field isolates and vaccine viruses in experimentally infected pregnant gilts. J. Gen. Virol..

[165] Zimmermann P., Ritzmann M., Selbitz H.J., Heinritzi K., Truyen U. (2006). VP1 sequences of German porcine parvovirus isolates define two genetic lineages. J. Gen. Virol..

[166] van Leengoed L.A., Vos J., Gruys E., Rondhuis P., Brand A. (1983). Porcine Parvovirus infection: Review and diagnosis in a sow herd with reproductive failure. Vet. Q..

[167] Xiao C.-T., Gerber P.F., Giménez-Lirola L.G., Halbur P.G., Opriessnig T. (2013). Characterization of porcine parvovirus type 2 (PPV2) which is highly prevalent in the USA. Vet. Microbiol..

[168] Gava D., Souza C.K., Schaefer R., Vincent A.L., Cantão M.E., Coldebella A., Ciacci-Zanella J.R. (2015). A TaqMan-based real-time PCR for detection and quantification of porcine parvovirus 4. J. Virol. Methods.

[169] Karniychuk U.U., Van Breedam W., Van Roy N., Rogel-Gaillard C., Nauwynck H.J. (2012). Demonstration of microchimerism in pregnant sows and effects of congenital PRRSV infection. Vet. Res..

[170] Ladinig A., Ashley C., Detmer S.E., Wilkinson J.M., Lunney J.K., Plastow G., Harding J.C.S. (2015). Maternal and fetal predictors of fetal viral load and death in third trimester, type 2 porcine reproductive and respiratory syndrome virus infected pregnant gilts. Vet. Res..

[171] Ladinig A., Wilkinson J., Ashley C., Detmer S.E., Lunney J.K., Plastow G., Harding J.C.S., Meng X.-J. (2014). Variation in fetal outcome, viral load and ORF5 sequence mutations in a large scale study of phenotypic responses to late gestation exposure to type 2 porcine reproductive and respiratory syndrome virus. PLoS ONE.

[172] Lager K.M., Halbur P.G. (1996). Gross and microscopic lesions in porcine fetuses infected with porcine reproductive and respiratory syndrome virus. J. Vet. Diagn. Investig..

[173] Rowland R.R.R., Lawson S., Rossow K., Benfield D.A. (2003). Lymphoid tissue tropism of porcine reproductive and respiratory syndrome virus replication during persistent infection of pigs originally exposed to virus in utero. Vet. Microbiol..

[174] Malgarin C.M., Nosach R., Novakovic P., Suleman M., Ladinig A., Detmer S.E., MacPhee D.J., Harding J.C.S. (2019). Classification of fetal resilience to porcine reproductive and respiratory syndrome (PRRS) based on temporal viral load in late gestation maternal tissues and fetuses. Virus Res..

[175] Joo H.S. (1999). Infectious Reproductive Diseases in Swine: Etiology and Clinical SIgns. Ph.D. Thesis.

[176] Kaur A., Mahajan V., Leishangthem G.D., Singh N.D., Bhat P., Banga H.S., Filia G. (2016). Epidemiological and immunopathological studies on Porcine parvovirus infection in Punjab. Vet. World.

[177] Novakovic P., Harding J.C.S., Al-Dissi A.N., Ladinig A., Detmer S.E., Leung F.C. (2016). Pathologic Evaluation of Type 2 Porcine Reproductive and Respiratory Syndrome Virus Infection at the Maternal-Fetal Interface of Late Gestation Pregnant Gilts. PLoS ONE.

[178] Madson D.M., Patterson A.R., Ramamoorthy S., Pal N., Meng X.J., Opriessnig T. (2009). Effect of porcine circovirus type 2 (PCV2) vaccination of the dam on PCV2 replication in utero. Clin. Vaccine Immunol..

[179] Brunborg I.M., Jonassen C.M., Moldal T., Bratberg B., Lium B., Koenen F., Schönheit J. (2007). Association of myocarditis with high viral load of porcine circovirus type 2 in several tissues in cases of fetal death and high mortality in piglets. A case study. J. Vet. Diagn. Investig..

[180] Oraveerakul K., Choi C.S., Molitor T.W. (1993). Tissue tropisms of porcine parvovirus in swine. Arch. Virol..

[181] Kranker S., Nielsen J., Bille-Hansen V., Bøtner A. (1998). Experimental inoculation of swine at various stages of gestation with a Danish isolate of porcine reproductive and respiratory syndrome virus (PRRSV). Vet. Microbiol..

[182] Hogg G.G., Lenghaus C., Forman A.J. (1977). Experimental porcine parvovirus infection of foetal pigs resulting in abortion, histological lesions and antibody formation. J. Comp. Pathol..

[183] Christianson W.T., Collins J.E., Benfield D.A., Harris L., Gorcyca D.E., Chladek D.W., Morrison R.B., Joo H.S. (1992). Experimental reproduction of swine infertility and respiratory syndrome in pregnant sows. Am. J. Vet. Res..

[184] Dvorak C.M.T., Yang Y., Haley C., Sharma N., Murtaugh M.P. (2016). National reduction in porcine circovirus type 2 prevalence following introduction of vaccination. Vet. Microbiol..

[185] Opriessnig T., McKeown N.E., Harmon K.L., Meng X.J., Halbur P.G. (2006). Porcine circovirus type 2 infection decreases the efficacy of a modified live porcine reproductive and respiratory syndrome virus vaccine. Clin. Vaccine Immunol..

[186] Zimmerman J.J., Yoon K.J., Wills R.W., Swenson S.L. (1997). General overview of PRRSV: A perspective from the United States. Vet. Microbiol..

[187] Saporiti V., Martorell S., Cruz T.F., Klaumann F., Correa-Fiz F., Balasch M., Sibila M., Segalés J. (2020). Frequency of Detection and Phylogenetic Analysis of Porcine circovirus3 (PCV-3) in Healthy Primiparous and Multiparous Sows and Their Mummified Fetuses and Stillborn. Pathogens.

[188] Afghah Z., Webb B., Meng X.-J., Ramamoorthy S. (2017). Ten years of PCV2 vaccines and vaccination: Is eradication a possibility?. Vet. Microbiol..

[189] Kumar N., Sharma S., Barua S., Tripathi B.N., Rouse B.T. (2018). Virological and immunological outcomes of coinfections. Clin. Microbiol. Rev..

[190] Salogni C., Lazzaro M., Giacomini E., Giovannini S., Zanoni M., Giuliani M., Ruggeri J., Pozzi P., Pasquali P., Boniotti M.B. (2016). Infectious agents identified in aborted swine fetuses in a high-density breeding area: A three-year study. J. Vet. Diagn. Investig..

[191] Serena M.S., Dibárbora M., Olivera V., Metz G.E., Aspitia C.G., Pereda A., Echeverría M.G., Cappuccio J. (2021). Evidence of porcine circovirus type 2 and co-infection with ungulate protoparvovirus 1 (porcine parvovirus) in mummies and stillborn piglets in subclinically infected farm. Infect. Genet. Evol..

[192] Jiang Y., Shang H., Xu H., Zhu L., Chen W., Zhao L., Fang L. (2010). Simultaneous detection of porcine circovirus type 2, classical swine fever virus, porcine parvovirus and porcine reproductive and respiratory syndrome virus in pigs by multiplex polymerase chain reaction. Vet. J..

[193] Zeng Z., Liu Z., Wang W., Tang D., Liang H., Liu Z. (2014). Establishment and application of a multiplex PCR for rapid and simultaneous detection of six viruses in swine. J. Virol. Methods.

[194] Tang Q., Ge L., Tan S., Zhang H., Yang Y., Zhang L., Deng Z. (2022). Epidemiological Survey of Four Reproductive Disorder Associated Viruses of Sows in Hunan Province during 2019-2021. Vet. Sci..

[195] Pescador C.A., Bandarra P.M., Castro L.A., Antoniassi N.A.B., Ravazzolo A.P., Sonne L., Cruz C.E.F., Driemeier D. (2007). Co-infection by porcine circovirus type 2 and porcine parvovirus in aborted fetuses and stillborn piglets in southern Brazil. Pesq. Vet. Bras..

[196] Mak C.K., Yang C., Jeng C.-R., Pang V.F., Yeh K.-S. (2018). Reproductive failure associated with coinfection of porcine circovirus type 2 and porcine reproductive and respiratory syndrome virus. Can. Vet. J..

[197] Ritzmann M., Wilhelm S., Zimmermann P., Etschmann B., Bogner K.H., Selbitz H.J., Heinritzi K., Truyen U. (2005). Prevalence and association of porcine circovirus type 2 (PCV2), porcine parvovirus (PPV) and porcine reproductive and respiratory syndrome virus (PRRSV) in aborted fetuses, mummified fetuses, stillborn and nonviable neonatal piglets. DTW Dtsch. Tierarztl. Wochenschr..

[198] Chen G.H., Mai K.J., Zhou L., Wu R.T., Tang X.Y., Wu J.L., He L.L., Lan T., Xie Q.M., Sun Y. (2017). Detection and genome sequencing of porcine circovirus 3 in neonatal pigs with congenital tremors in South China. Transbound. Emerg. Dis..

