# Supplementary material for: Effects of Primary Viruses (PCV2, PPV1, and PRRSV) Involved in Porcine Reproductive Failure as Mono- and Coinfections with Each Other and with Emerging Viruses (PCV3 and nPPVs)
Source: Viruses. 2025 Aug 19;17(8):1137. doi: 10.3390/v17081137 (PMC12390666; doi:10.3390/v17081137)
Supplement: Supplementary file 1 [file viruses-17-01137-s001.zip › viruses-3737747-supplementary.pdf]

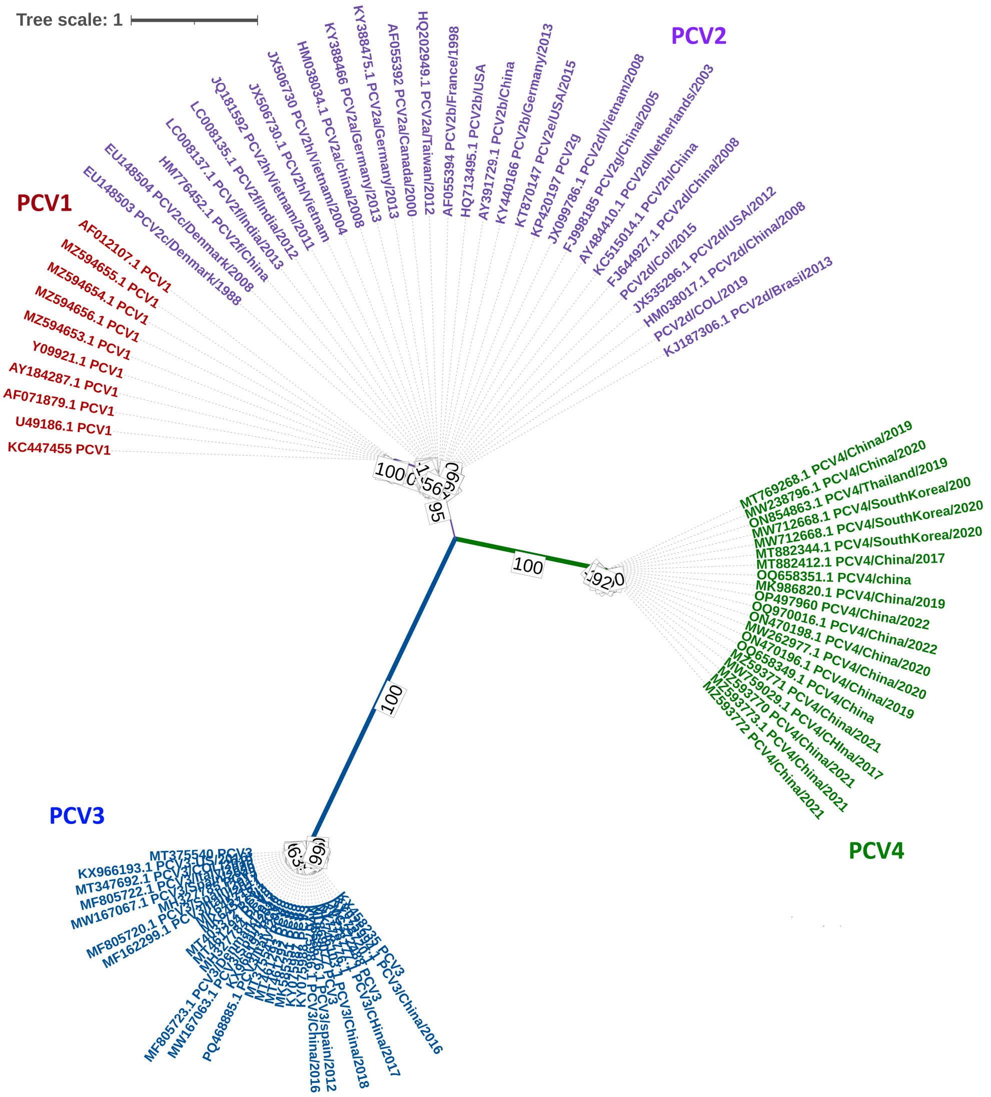

Figure S1. Phylogenetic distribution of porcine circoviruses (PCVs) based on the alignment of 50 representative whole-genome nucleotide (nt) sequences from PCV1 to PCV4, retrieved from the NCBI Genbank nt database. The phylogenetic tree was constructed using the Neighbor-joining method and the p-distance model. The analysis was performed using MEGA - version 11.0 [49]. Boostrap values are indicated on the branches.

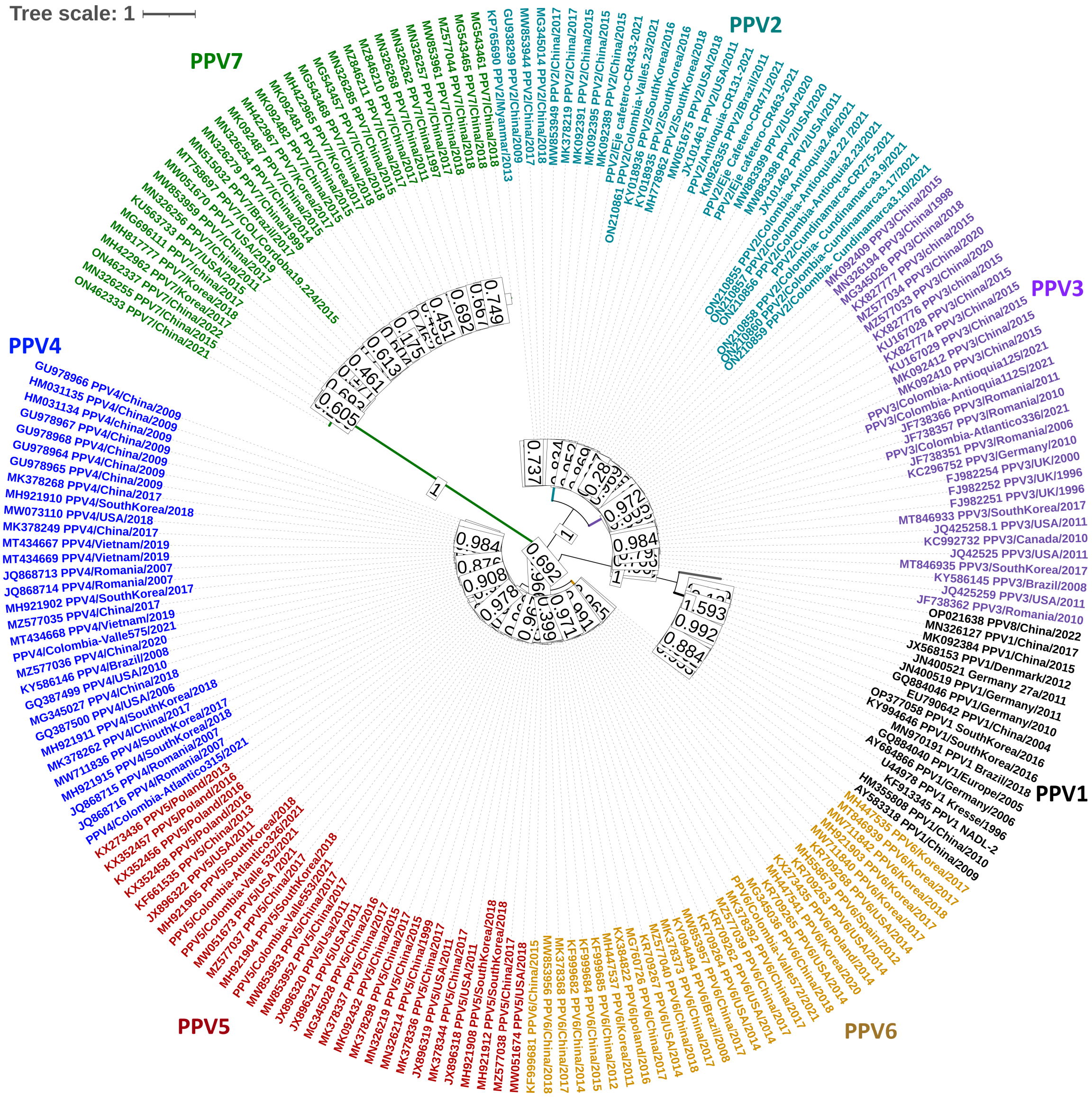

Figure S2. Phylogenetic distribution of porcine parvoviruses (PPV1 through PPV8). This distribution was determined by aligning 200 representatives nonstructural (NS) gene sequences retrieved from the NCBI GenBank nucleotide database. The phylogenetic tree was constructed using the Neighbor-joining method with the p-distance model. Boostrap values are indicated by grey circles. The analysis was performed using MEGA - version 11.0 [49]. Boostrap values are indicated on the branches.

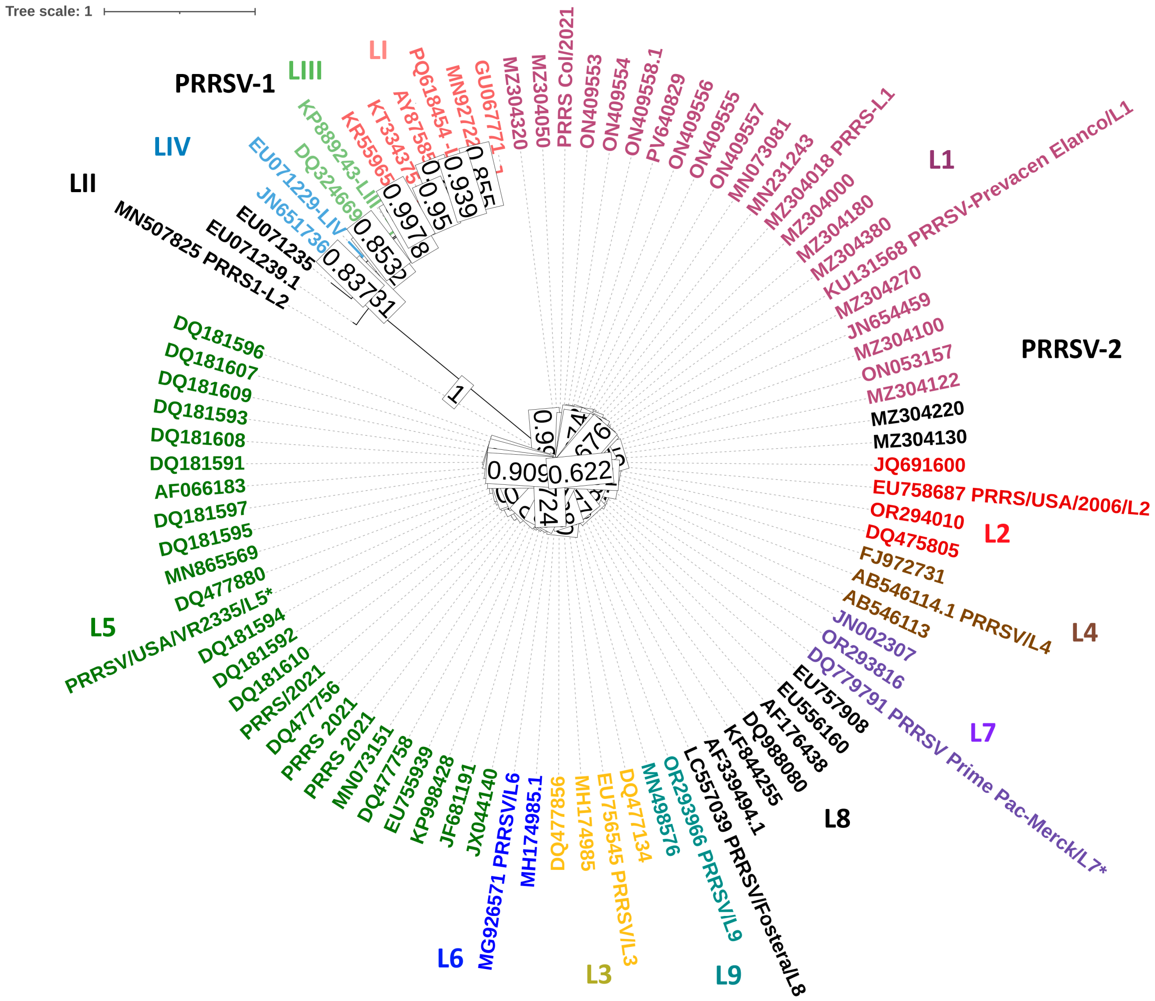

Figure S3. Phylogenetic distribution of porcine reproductive and respiratory syndrome virus (PRRSV). This analysis is based on the alignment of 80 representative ORF5 nucleotide (nt) sequences from PRRSV-1 and PPRSV-2, which are available in the NCBI GenBank nt database. The phylogenetic tree was constructed using the Neighbor-joining method along with the K2+G+I model. Boostrap values are indicated on the branches. The analysis was performed using MEGA - version 11.0 [49].
